# Heterologous SARS-CoV-2 spike protein booster elicits durable and broad antibody responses against the receptor-binding domain

**DOI:** 10.1038/s41467-023-37128-1

**Published:** 2023-03-15

**Authors:** Tomohiro Takano, Takashi Sato, Ryutaro Kotaki, Saya Moriyama, Shuetsu Fukushi, Masahiro Shinoda, Kiyomi Kabasawa, Nagashige Shimada, Mio Kousaka, Yu Adachi, Taishi Onodera, Kazutaka Terahara, Masanori Isogawa, Takayuki Matsumura, Masaharu Shinkai, Yoshimasa Takahashi

**Affiliations:** 1 Research Center for Drug and Vaccine Development, National Institute of Infectious Diseases, Tokyo, 162-8640 Japan; 2 Tokyo Shinagawa Hospital, Tokyo, 140-8522 Japan; 3 Department of Virology I, National Institute of Infectious Diseases, Tokyo, 162-8640 Japan

**Keywords:** SARS-CoV-2, Vaccines

## Abstract

The immunogenicity of mRNA vaccines has not been well studied when compared to different vaccine modalities in the context of additional boosters. Here we show that longitudinal analysis reveals more sustained SARS-CoV-2 spike receptor-binding domain (RBD)-binding IgG titers with the breadth to antigenically distinct variants by the S-268019-b spike protein booster compared to the BNT162b2 mRNA homologous booster. The durability and breadth of RBD-angiotensin-converting enzyme 2 (ACE2) binding inhibitory antibodies are pronounced in the group without systemic adverse events (AEs) after the S-268019-b booster, leading to the elevated neutralizing activities against Omicron BA.1 and BA.5 variants in the stratified group. In contrast, BNT162b2 homologous booster elicited antibodies to spike N-terminal domain in proportion to the AE scores. High-dimensional immune profiling identifies early CD16^+^ natural killer cell dynamics with CCR3 upregulation, as one of the correlates for the distinct anti-RBD antibody responses by the S-268019-b booster. Our results illustrate the combinational effects of heterologous booster on the immune dynamics and the durability and breadth of recalled anti-RBD antibody responses against emerging virus variants.

## Abstract (web summary)

Takano et al. show that heterologous booster by SARS-CoV-2 recombinant spike protein vaccine recalls a more sustained and broader anti-spike receptor-binding domain antibody response compared to homologous booster by mRNA vaccine.

## Introduction

The rapid and continuous evolution of severe acute respiratory syndrome coronavirus 2 (SARS‑CoV‑2), leading to the emergence of variants of concern^1–7^, poses a great challenge in the development of vaccines that elicit a broad antibody response. Moreover, antibody waning is associated with a reduction in vaccine effectiveness over time^8–11^, highlighting the need for a vaccine regimen to elicit a durable antibody response also. While the variants-adapted vaccination campaigns are ongoing, as the U.S. Food and Drug Administration recommends booster shots for Omicron subvariants^12^, it is also important to prepare the vaccines that induce broad and sustained antibody responses. The durability and breadth of antibody responses are regulated by cellular networks consisting of diverse types of immune cells that are activated by the vaccination. The systems vaccinology approaches have unveiled the early innate immune cell signatures, including natural killer (NK)/monocyte subsets, that correlate with antibody responses after booster vaccinations^13–17^. In line with the findings of other groups, we have previously identified mRNA vaccine-induced immune cell dynamics that correlate with antibody responses at the peak time point^18^. However, it is unclear whether these correlates are applied in antibody responses triggered by other modalities of vaccine, related to the durability and breadth to variants. Determining the immunological features elicited by heterologous booster is useful for designing the vaccine strategy.

The BNT162b2 mRNA vaccine in a two-dose setting induces robust neutralizing antibody responses against SARS-CoV-2 with a 95% efficacy in preventing coronavirus disease 2019 (COVID-19)^19^. Besides the mRNA vaccines, other COVID-19 vaccines have been developed worldwide using different modalities, such as viral vectors, inactivated whole virions, and recombinant protein formulations^20–23^. However, no superiority of antibody responses in viral vector, inactivated, and recombinant protein vaccines compared to mRNA vaccine has been demonstrated for primary series vaccination^24–26^.

S-268019-b is a novel vaccine candidate comprising a modified full-length recombinant SARS-CoV-2 spike protein S-910823 produced by the baculovirus expression system and is combined with a squalene-based oil-in-water emulsion adjuvant A-910823^27–30^. S-910823 is the antigen based on amino acid sequences of full-length spike protein in Wuhan-Hu-1 isolate with a mutation in the furin cleavage site to inhibit cleavage between S1 and S2 subunits of the spike protein and with the substitution of two proline residues to improve prefusion conformation stability of the spike protein trimer^31–33^. The protein vaccines, including S-268019-b, are combined with adjuvants that greatly enhance the magnitude of the subsequent immune response. Indeed, a randomized, observer-blinded, phase 2/3 study has shown that the use of the S-268019-b recombinant protein vaccine as a booster (third) dose is noninferior in inducing neutralizing antibodies on day 28 post-vaccination and leads to a lower incidence of adverse events (AEs) compared to the BNT162b2 mRNA vaccine^29^.

In this study, we aim to profile the immune responses after the heterologous booster by the S-268019-b vaccine by applying high-dimensional serological and flow cytometric analysis. This is an observational study, relating yet separate from the clinical trial in terms of the samples and research objectives^29^. The participants are voluntarily recruited based on separate content. Here, we show that longitudinal analysis reveals more sustained SARS-CoV-2 spike receptor-binding domain (RBD)-binding IgG titers with the breadth to antigenically distinct variants by the S-268019-b spike protein booster compared to the BNT162b2 mRNA homologous booster.

## Results

### Study population

**Fig. 1:**
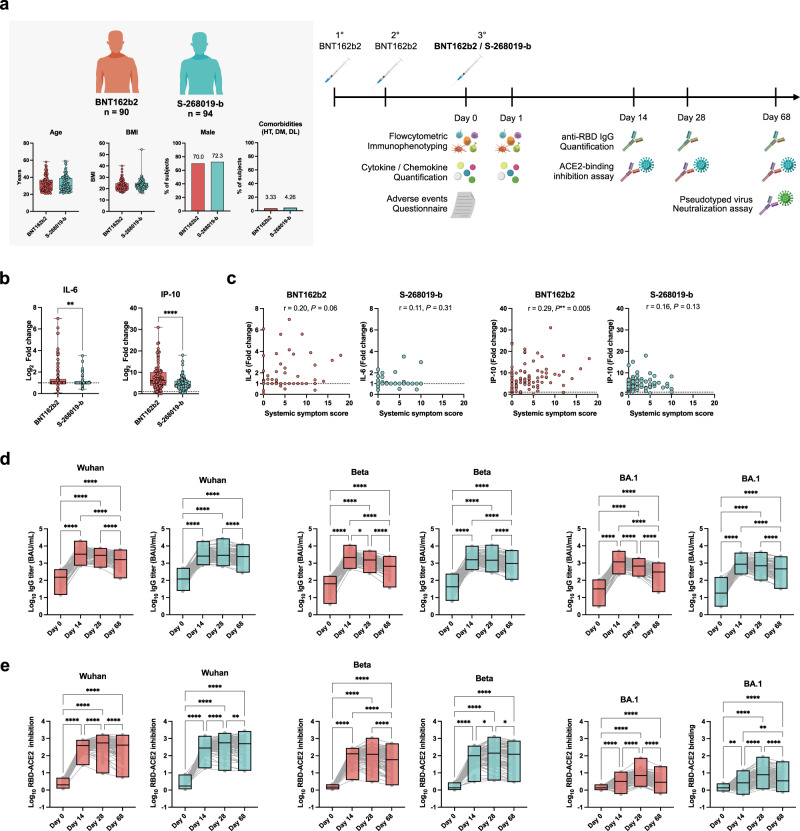
Adverse events, plasma cytokine dynamics, and antibody kinetics following the third vaccination. **a** Summary of the cohort demographics: age, body mass index (BMI), male ratio, and comorbidities (left). HT, hypertension; DM, diabetes mellitus; DL, dyslipidemia. A cohort of healthcare workers who received two doses of BNT162b2 (*n* = 184) was evaluated for antibody responses, incidence of adverse events, immune cell dynamics, and cytokine/chemokine production (right). **b** Fold changes (Day 1 concentration/Day 0 concentration) of IL-6 and IP-10 concentrations. **c** Correlations between the systemic symptom severity score and IL-6 or IP-10. **d** receptor-binding domain (RBD)-binding IgG titers to Wuhan, Beta, and Omicron BA.1 strains in plasma were longitudinally quantified. **e** Relative RBD- angiotensin-converting enzyme 2 (ACE2) inhibition to Wuhan, Beta, and Omicron BA.1 strains in plasma were longitudinally quantified. For box and whisker plots, the whisker indicates minimum and maximum, and the box indicates the 25th and 75th percentiles (edges of the box), and median (center line) (**a**, **b**). The box plot indicates minimum, maximum, and median (**d**, **e**). Each dot represents the data from each individual (**a**–**c**) and dots connected with lines indicate data from the same individuals (**d**, **e**). Statistical analyses were performed using the two-tailed Mann–Whitney test (**b**), Spearman’s rank order coefficient (**c**), and Friedman test (**d**, **e**) [(**b**–**e**) *P < 0.05, **P < 0.01, ***P < 0.001, and ****P < 0.0001]. Data were pooled from ≥2 independent experiments [BNT162b2 (red), *n* = 90; S-268019-b (blue), *n* = 94]. Source data are provided as a Source Data file.

Blood samples and AE questionnaires were collected for this study from 184 volunteers who participated in the Phase 2/3 trial of the S-268019-b vaccine^29^. The participants were voluntarily recruited by separate consent for this observational study. All participants were confirmed to be uninfected by SARS-CoV-2 during the study period, as determined by the anti-nucleocapsid antibody in pre- and post-vaccinated plasma. They were all vaccinated with a second dose of BNT162b2 ≥ 6 months prior. For their third dose, 90 individuals received BNT162b2, and 94 received S-268019-b (Fig. 1a). In the BNT162b2 and S-268019-b groups, the median age was 31 (interquartile range [IQR]: 25–37 years) and 30 years (IQR: 25–39 years), median body mass index (BMI) was 21.5 (IQR: 19.4–24.7) and 22.7 (IQR: 20.7–25.1), male population was 70.0% and 72.3% of the total population, and the prevalence of comorbidities (hypertension, diabetes mellitus, and dyslipidemia) was 3.33% and 4.26%, respectively (Fig. 1a). Baseline demographics and participant characteristics were balanced across the two groups.

### S-268019-b booster increases plasma IL-6 and IP-10 at lesser extents than BNT162b2 booster

Compared to BNT162b2, the S-268019-b booster leads to a lower incidence of treatment-related AEs^29^. The 48 regulatory cytokines and chemokines potentially involved in inflammatory responses were quantified in the plasma. Their fold changes prior to and 1 day after the booster were calculated (Fig. 1b and Supplementary Fig. 1). Higher fold increases of IL-6 and IP-10 concentrations were observed in the BNT162b2 booster group (Fig. 1b). There was significant correlation between increased IP-10 concentration and the systemic symptom scores (Fig. 1c and Supplementary Table 1). While, in the S-268019-b group, there was no correlation between slightly increased IL-6 and IP-10 concentrations and systemic symptom scores (Fig. 1c and Supplementary Table 1). Therefore, systemic inflammation mediated by increased IL-6 and IP-10 concentrations may account for higher symptom scores in the BNT162b2 booster group but not in the S-268019-b group.

### S-268019-b booster elicits more sustained antibody responses than the BNT162b2 booster

The SARS-CoV-2 spike RBD-binding IgG titer and the inhibitory activity on RBD-angiotensin-converting enzyme 2 (ACE2) binding, a surrogate for neutralizing titer^34–37^, were both longitudinally assessed using an electrochemiluminescence immunoassay (ECLIA)^29^. The booster doses of both vaccines induced similar antibody responses on day 14, based on the binding to Wuhan RBDs (Fig. 1d) and RBD-ACE2 binding inhibitory activity (Fig. 1e). The Wuhan antibody responses peaked on day 14 or 28 and gradually declined by day 68. The responses to Beta and Omicron BA.1 RBDs followed similar kinetics; however, the overall RBD-ACE2 binding inhibitory activity against Omicron BA.1 was at lower magnitudes.

**Fig. 2:**
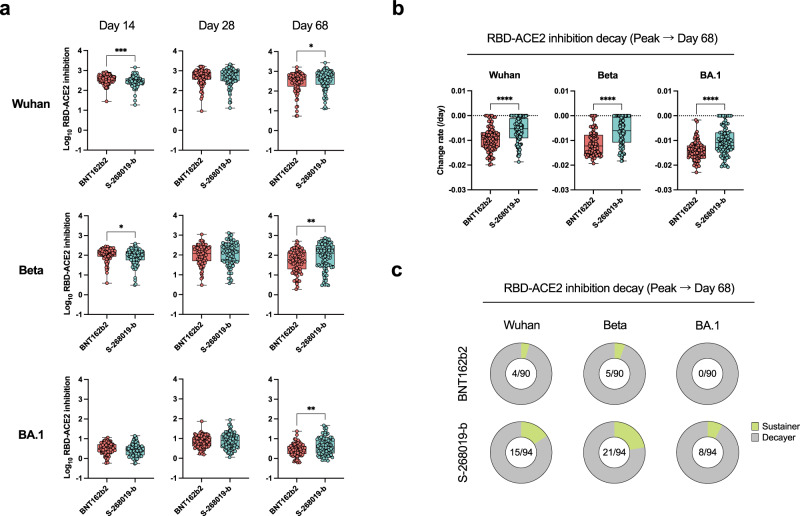
Longitudinal analyses of the inhibitory activity on RBD-ACE2 binding following the third vaccination. **a** Relative receptor-binding domain (RBD)-angiotensin-converting enzyme2 (ACE2) inhibition to the variants on days 14, 28, and 68. **b** Decay rate of the relative RBD-ACE2 inhibition to the variant RBDs from the peak to day 68 after the third vaccination. **c** Ratios of the sustainers and decayers for relative RBD-ACE2 inhibition. For box and whisker plots, the whisker indicates minimum and maximum, and the box indicates the 25th and 75th percentiles (edges of the box), and median (center line) (**a**, **b**), and each dot represents the data from individuals (**a**, **b**). Statistical analyses were performed using the two-tailed Mann–Whitney test [(**a**, **b**) *P < 0.05, **P < 0.01, ***P < 0.001, and ****P < 0.0001]. Data were pooled from ≥2 independent experiments [BNT162b2 (red), *n* = 90; S-268019-b (blue), *n* = 94]. Source data are provided as a Source Data file.

The durability of the antibody responses elicited by the two vaccines was compared at the peak (days 14 and 28) and the contraction phase (day 68). Given the 3.9-fold decline of antibody responses by days 43–70 after the booster dose of mRNA vaccine^38^, 68 days are sufficiently long to visualize the antibody decay. Compared to the S-260819-b booster group, RBD-binding IgG titers (1.1-fold) and RBD-ACE2 binding inhibitory activities (1.2-fold) were slightly higher in the BNT162b2 booster group on day 14; however, such difference was no longer detected on day 28. Conversely, the S-268019-b-elicited responses were higher at day 68, demonstrating a milder decay of the antibody responses in the S-268019-b booster group (Supplementary Fig. 2a and Fig. 2a). Indeed, the titer decay rates from the peak to day 68 clearly showed more sustained responses in the S-268019-b booster to the Beta and Omicron BA.1 variants (Supplementary Fig. 2b and Fig. 2b). Furthermore, we observed a higher ratio of sustainers that did not show any antibody decline during the observation period in the S-268019-b booster group (Fig. 2c). Together, S-268019-b booster elicited more sustained antibody responses, especially against Beta and Omicron BA.1 variants, at least up to day 68.

The breadth of RBD-binding IgG titers and RBD-ACE2 binding inhibitory activities against the variants was evaluated by the relative IgG titer and RBD-ACE2 inhibition to the Wuhan strain versus the Beta/Omicron BA.1 variants. Although any differences were not found in the breadth between the BNT162 b2 and S-268019-b booster groups on days 14 and 28, the breadth on day 68 was significantly higher in both RBD-binding IgG titers and RBD-ACE2 binding inhibitory activities in the S-268019-b booster group (Supplementary Fig. 2d, e). Using available blood samples, the durability and breadth of RBD-binding IgG titers and RBD-ACE2 binding inhibitory activities were further evaluated on day 182. The results confirmed more sustained antibody responses in the S-268019-b vaccine group than in the BNT162b2 group on day 182 (Supplementary Fig. 2f, g). Hereafter, further analyses on the contraction phase were performed using day 68 samples, owing to the availability of a larger sample size.

### S-268019-b booster induces sustained antibody responses even in those who show no systemic AEs

**Fig. 3:**
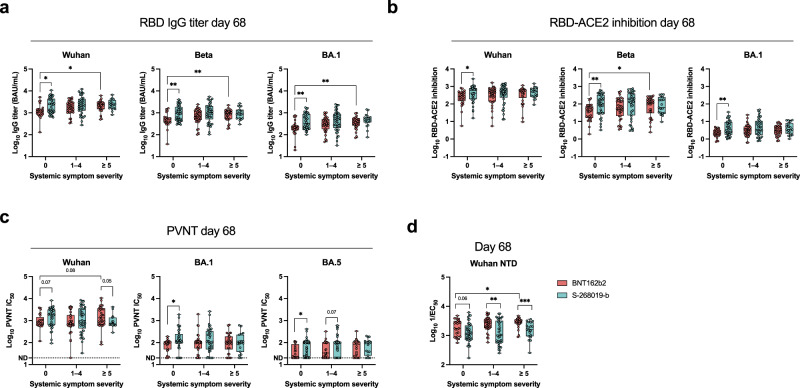
Plasma antibody responses in vaccinees stratified by systemic symptom severity scores. Receptor-binding domain (RBD)-binding IgG titers (**a**) and relative RBD-angiotensin-converting enzyme 2 (ACE2) inhibition (**b**) to the variant RBDs on day 68. Neutralization half-maximal inhibition concentration (IC_50_) (**c**) of the variant pseudotyped viruses, and N-terminal domain (NTD)-binding IgG titers (**d**) on day 68. For box and whisker plots, the whisker indicates minimum and maximum, and the box indicates the 25th and 75th percentiles (edges of the box), and median (center line). Each dot represents data from individuals. Statistical analyses were performed using the Kruskal–Wallis test followed by Dunn’s post hoc test (comparisons among the groups stratified by systemic symptom severity scores within the same vaccine group) or the two-tailed Mann–Whitney test [comparisons between BNT162b2 (red) and S-268019-b (blue) groups within the same systemic symptom severity] (*P < 0.05 and **P < 0.01). Data were pooled from ≥2 independent experiments [BNT162b2: *n* = 25 (score 0), *n* = 35 (score 1–4), and *n* = 30 (score ≥ 5); S-268019-b: *n* = 38 (score 0), *n* = 37 (score 1–4), and *n* = 19 (score ≥ 5)]. Source data are provided as a Source Data file.

We and other groups have previously noted positive correlations between the elicited antibody titers and systemic AEs after two doses of BNT162b2 vaccines^18,39–43^. Stratification of this cohort by systemic symptom scores (0: *n* = 25; 1–4: *n* = 35; ≥5: *n* = 30) revealed higher IgG titers on day 68 in those who had higher symptom scores in the BNT162b2 group (Fig. 3a). A similar tendency was observed in RBD-ACE2 binding inhibitory activities against the variants (Fig. 3b). The peak antibody titers (days 14 and 28) failed to generate a such correlation between antibody and systemic symptom in the BNT162b2 group (Supplementary Fig. 3a–d), suggesting the contribution of antibody persistence rather than the levels of antibody peak. In contrast to the BNT162b2 results, the S-268019-b booster group comparably induced and sustained IgG antibodies on day 68, irrespective of systemic symptom scores (0: *n* = 38; 1–4: *n* = 37; ≥5: *n* = 19). The distinct antibody responses between the BNT162b2 and S-268019-b groups were evident in IgG titers and RBD-ACE2 inhibition of the participants without systemic symptoms (Fig. 3a, b). Concordantly, the increase in the antibody breadth on day 68 was more evident in the individuals with low symptom scores (Supplementary Fig. 3e, f). The antibody decay analysis within the stratified subgroups further confirmed more sustained antibody responses in the S-268019-b group without systemic symptom scores (Supplementary Fig. 3g, h). In addition, IgG titers against spike protein were also higher in the S-268019-b group when vaccinees without AEs were compared (Supplementary Fig. 3i), indicating whole antibody responses are more sustained in the S-268019-b group without AEs.

To examine the effect of the broad and durable anti-RBD antibody responses by a booster vaccination with adjuvanted protein on the overall neutralizing antibody responses, we quantified neutralizing antibody titers on day 68 using three pseudotyped viruses expressing Wuhan, Omicron BA.1, and BA.5 spike proteins, respectively. Similar to the results from the RBD-ACE2 inhibition, the neutralizing titers to Wuhan (*P* = 0.07), Omicron BA.1 (*P* < 0.05), and BA.5 (*P* < 0.05) were elevated in the S-268019-b group compared to the BNT162b2 group among the vaccinees without any systemic AEs (Fig. 3c). The neutralization titer and the inhibitory activity on RBD-ACE2 binding indeed correlated each other, confirming the rational for utilizing RBD-ACE2 inhibition titers as surrogates of neutralization titers in this cohort (Supplementary Fig. 3j, k). However, despite the overall increase in RBD-ACE2 inhibitory activities on day 68, the neutralizing titers failed to generate a significant difference between the two vaccine groups when all the vaccinees were included for the analysis without stratification by AEs (Supplementary Fig. 4a).

To gain insights into the dissociation between RBD-ACE2 inhibitory activities and neutralizing titers, IgG titers against spike N-terminal domain (NTD) were also quantitated. We observed higher anti-NTD IgG titers in the BNT162b2 group compared to the S-268019-b group (Supplementary Fig. 4b). The elevation of anti-NTD IgG titers was most prominent in the BNT162b2 vaccinees with high symptom scores (≥ 5) (Fig. 3d), and such elevation was less pronounced in those without AEs. Since a fraction of anti-NTD antibodies exhibits neutralizing activity^44–46^, the higher NTD response in the BNT162b2 group may compensate for the neutralization activity by anti-RBD antibodies.

**Fig. 4:**
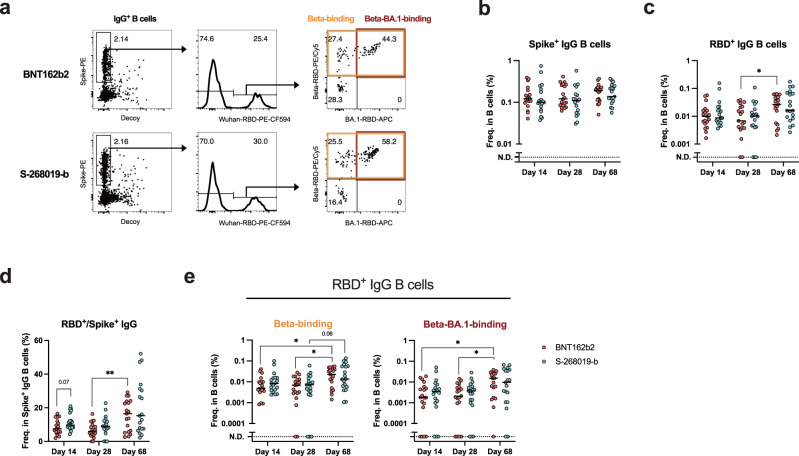
Plasma antibody and B_mem_ responses in vaccinees without systemic adverse events. **a** Representative plots showing the analyses of IgG^+^ B cells binding to spike and receptor-binding domain (RBDs). Frequencies of spike^+^ IgG B cells (**b**) and RBD^+^ IgG B cells (**c**) in the B cells. Frequencies of the RBD^+^ IgG B cells in the spike^+^ IgG B cells (**d**) and the variants RBD^+^ IgG B cells in the B cells (**e**). For box and whisker plots, the whisker indicates minimum and maximum, and the box indicates the 25th and 75th percentiles (edges of the box), and median (center line) (**b**–**e**). Each dot represents data from individuals (**b**–**e**). Statistical analyses were performed using the Kruskal–Wallis test followed by Dunn’s post hoc test (comparisons among the different time points) or the two-tailed Mann–Whitney test [comparisons between BNT162b2 (red) and S-268019-b (blue) groups within the same time point] [(**b**–**e**) *P < 0.05 and **P < 0.01]. Data were pooled from ≥2 independent experiments [(**b**–**e**) BNT162b2: *n* = 19; S-268019-b: *n* = 19]. Source data are provided as a Source Data file.

Humoral immune responses induced by vaccination include antibodies, which we have assessed above, and memory B cells. We examined memory B cell responses induced by booster vaccination with BNT162b2 and S-268019-b without AEs. Longitudinal memory B cell frequency and breadth on days 14, 28, and 68 were assessed in the vaccinees without any systemic symptom scores who were selected without any biases on age, sex, and antibody reactivities (BNT162b2, *n* = 19; S-268019-b, *n* = 19) (Supplementary Fig. 5a, b). S-268019-b induced comparable IgG^+^ memory B cell responses against spike and RBD after BNT162b2 and S-268019-b booster (Fig. 4a–d, Supplementary Fig. 5c). The breadth to the Beta and BA.1 variants were also comparable among two vaccines (Fig. 4e). Collectively, we concluded that the S-268019-b booster induced fewer AEs, more sustained anti-RBD antibody responses, and comparable memory B cell responses compared with BNT162b2.

### S-268019-b booster triggers NK subset dynamics in correlation with the inhibitory activity on RBD-ACE2 binding

**Fig. 5:**
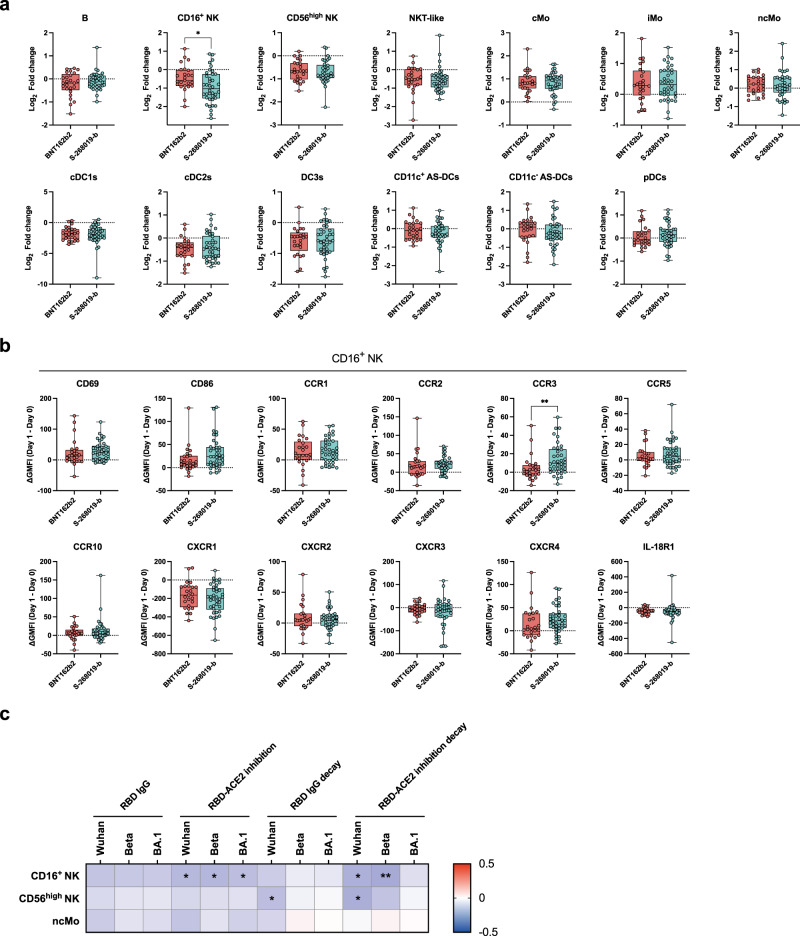
Immune cell dynamics correlating with the durability of antibody responses. **a** Post/pre ratios of B cells, CD16^+^ NK cells, CD56^high^ NK cells, NKT-like cells, classical monocytes, intermediate monocytes, non-classical monocytes, conventional DC1 (cDC1s), cDC2s, DC3s, CD11c^+^ AS-DCs, CD11c^-^ AS-DCs, and pDCs in the groups without systemic symptoms. **b** Differences in the CD69, CD86, CCR1, CCR2, CCR3, CCR5, CCR10, CXCR1, CXCR2, CXCR3, CXCR4, and IL-18R1 expression levels on CD16^+^ NK cells between days 1 and 0 in the groups without systemic symptoms. **c** Spearman’s rank coefficient correlations between antibody responses and immune cell dynamics in all samples after a 3rd vaccination. The color represents the correlation coefficient. For box and whisker plots, the whisker indicates minimum and maximum, and the box indicates the 25th and 75th percentiles (edges of the box), and median (center line) (**a**, **b**). Each dot represents data from individuals (**a**, **b**). Statistical analyses were performed using the two-tailed Mann–Whitney test (**a**, **b**) and Spearman’s rank order coefficient (**c**) (*P < 0.05 and **P < 0.01). Data were pooled from six independent experiments [(**a**, **b**) BNT162b2 (red): *n* = 25; S-268019-b (blue), *n* = 38; (**c**) BNT162b2: *n* = 90; S-268019-b: *n* = 94]. Source data are provided as a Source Data file.

To gain mechanistic insights into how the S-268019-b booster induces sustained and broad anti-RBD antibody responses, thirteen immune cell dynamics in the participants without systemic symptom scores were quantified (Supplementary Fig. 6a) by high-dimensional flow cytometry analysis, as previously reported^18^. The frequency of each cell subset was quantified as a percentage of the CD45^+^ cells, and then the fold increases/decreases from day 0 to day 1 were defined as cell dynamics hereafter. Among the 13 cell dynamics tested in this study, we found a more profound decrease in CD16^+^ NK cell dynamics in the S-268019-b group (Fig. 5a). In contrast, other cell dynamics, including CD56^high^ NK, non-classical monocyte (ncMo), and NKT-like cells, did not exhibit any differences between the two vaccine groups. The rapid disappearance of the CD16^+^ NK cells from circulation could be due to the prompt activation and chemotactic response to the chemokines secreted upon the booster vaccination. Therefore, the expression levels of two activation markers (CD69 and CD86) and ten chemokine receptors (CCR1, CCR2, CCR3, CCR5, CCR10, CXCR1, CXCR2, CXCR3, CXCR4, and IL-18R1) on the CD16^+^ NK cells were quantitated using the same flow cytometry analysis (Fig. 5b and Supplementary Fig. 6b). Of these, only CCR3 was upregulated more drastically in the S-268019-b group, suggesting the possible contribution of the CCR3 ligand/CCR3 axis to the rapid disappearance of CD16^+^ NK cells from peripheral blood. Finally, the possible link between CD16^+^ NK cell dynamics and the inhibitory activities on RBD-ACE2 binding was estimated using the correlation analysis (Fig. 5c). The CD16^+^ NK cell dynamics were inversely correlated with the RBD-ACE2 binding inhibitory activities and the decay from the peak to day 68 after the S-268019-b booster vaccination; in contrast, CD56^high^ NK cell and non-classical monocyte dynamics, correlates for antibody titers after the second dose of the BNT162b2 vaccine^18^, failed to show such correlation, except for the antibody titers to Wuhan in the CD56^high^ NK cells.

## Discussion

**Fig. 6:**
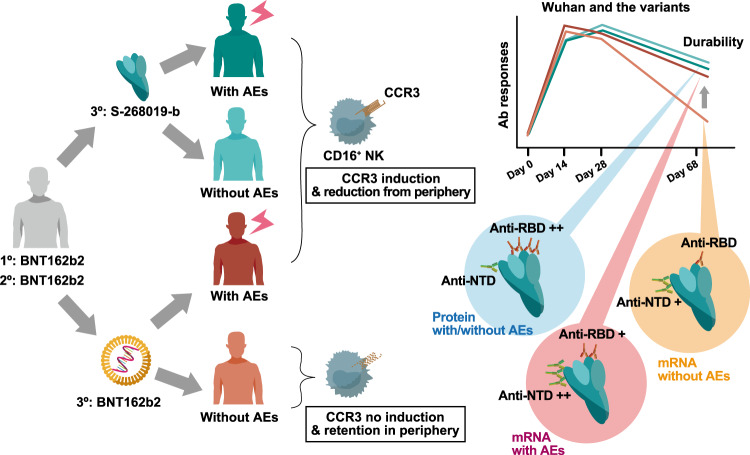
Summary of our findings. In immunocompetent adults who had received two doses of BNT162b2 vaccine, heterologous booster vaccination with S-268019-b vaccine upregulates CCR3 expression on CD16^+^ NK cells and facilitates their migration from peripheral blood on day 1 after vaccination regardless of adverse events (AEs) intensities. Such cell dynamics are associated with durable and broad plasma anti-receptor-binding domain (RBD) antibody responses. Homologous booster vaccination with BNT162b2 vaccine, when accompanied by AEs, elicits durable and broad plasma anti-RBD antibody responses similar to heterologous S-268019-b vaccine booster; however, homologous BNT162b2 vaccine booster without AEs fails to elicit CCR3 expression on CD16^+^ NK cells, migration of such cell subset from peripheral blood, and subsequent durable and broad anti-RBD antibody responses. BNT162b2 booster highly elicited anti-N-terminal domain (NTD) antibody responses compared to S-268019-b vaccine. The enhancement of anti-NTD antibody responses was most prominent by BNT162b2 vaccine booster with AEs and was less pronounced by BNT162b2 booster without AEs.

Heterologous prime-boost strategies for COVID-19 vaccines are currently being investigated worldwide^47,48^. However, the booster effects of recombinant protein vaccines have not yet been fully elucidated. The utilization of the S-268019-b recombinant protein vaccine as a heterologous booster on BNT162b2 mRNA vaccinees results in a similar level of neutralizing antibody responses at the peak and lower reactogenicity levels compared to the BNT162b2 mRNA vaccine^29^. The longitudinal analysis in this study revealed more sustained inhibitory activities on ACE2-binding to the RBD of Beta and Omicron BA.1 variants, which have distinct antigenicity, and provided new insights (Fig. 6). Remarkably, sustained and broad anti-RBD antibody responses after the S-268019-b booster were most prominent in those with fewer systemic symptom scores, suggesting less dependence on the inflammatory responses triggered by S-268019-b booster vaccination. Similar to the results from the RBD-ACE2 inhibition, the neutralizing titers against Omicron BA.1 and BA.5 variants were elevated in the S-268019-b group compared to the BNT162b2 group among the vaccinees without any systemic AEs. Although the neutralizing antibody titers between the two vaccine groups were comparable without stratification by AEs, the results probably reflect higher anti-NTD IgG titers in the BNT162b2 group with high symptom scores. High-dimensional immune profiling further dissected the early CD16^+^ NK cell dynamics as a correlate of such distinct anti-RBD antibody responses in the S-268019-b booster. The current study still has a few limitations. (1) Our study does lack data related to protection. It remains undetermined the extents to which durable and broad antibody responses together with cellular immunity contribute to preventing infection or severe diseases. (2) Our analysis is based on a small cohort size. It is important to accumulate data in larger studies before generalizing the present results. Under the limitations, we revealed that heterologous mRNA/recombinant protein vaccination strategy could induce more sustained and broader antibody responses with a preference for the receptor-binding domain, probably by activating fewer inflammatory immune cell dynamics. The findings also suggest the feasibility of a heterologous vaccination strategy against COVID-19, which could speed up the global vaccination campaign and maximize pandemic control.

We identified higher IL-6 and IP-10 levels in the BNT162b2 group, compared to the S-268019-b group. Lipid nanoparticles trigger IL-6 secretion in a murine model, which is required for efficient Tfh induction and is important for subsequent germinal center reactions^49^. IP-10 is also one of the signatures associated with the effective immune response to SARS-CoV-2 in BNT162b2 vaccines^50^. Therefore, IL-6 and IP-10 would potentiate the immunogenicity of mRNA vaccines, but the elevated production of these cytokines/chemokines may also exaggerate adverse reactions, as reported in a previous study on adjuvanted hepatitis B vaccine^51^. In contrast, in the S-268019-b group, slight increases in IL-6 and IP-10 concentrations were not associated with systemic symptom scores or durable and broad anti-RBD antibody responses. Lower levels of IL-6 and IP-10 induction after the S-268019-b booster might account for its lower reactogenicity when compared to the BNT162b2 booster. Furthermore, the robust antibody recall by the S-268019-b booster suggests the involvement of pathways other than the IL-6/IP-10-mediated inflammatory signals.

Both antigen and adjuvant could play roles in the sustained and broad anti-RBD antibody responses by the S-268019-b booster. Probably, the purified recombinant protein antigen could retain a relatively uniform antigenic structure, whereas mRNA vaccines might synthesize the relatively crude antigen in a host cell-dependent manner, thereby increasing the potential difference and variability in the antigenic structure. Therefore, among the memory B cell repertoires primed by the BNT162b2-derived antigens, those recalled by the S-268019-b are expected to focus on the epitopes that are conserved among the two vaccine antigens. Such a prime-boost regime may be relevant to the broader antibody responses of a heterologous booster than a homologous booster. In addition, our results showed that the BNT162b2 booster induced higher anti-NTD IgG titers than the S-268019-b booster. Epitope dominancy of B cell recall responses against the same antigen is affected by pre-existing antibodies induced by the prior responses, called “antibody feedback”^52–54^. The pre-existing antibodies may have masked the RBD epitopes focused by BNT162b2 priming and drifted epitope preference toward other sites including NTD at the homologous BNT162b2 booster. Heterologous booster by S-268019-b might be less affected by the antibody feedback since distinct vaccine modalities have distinct epitope preferences on RBD responses^55^. Our results also showed that the homologous and heterologous booster induced comparable IgG titer against spike protein among the vaccinees with AEs, whereas the titer was lower in BNT162b2-boosted vaccinees without AEs. Collectively, the S-268019-b booster responses are different from the homologous BNT162b booster in that the responses have a preference for RBD with magnitudes irrelevant to AEs.

Besides the difference in the antigen, the adjuvant possibly accounts for the sustained and broad anti-RBD responses induced by the S-268019-b booster. The S-268019-b vaccine includes the squalene-based adjuvant A-910823. Although the molecular mechanisms by which the A-910823 adjuvants work in humans are not well understood, several studies have reported the induction of persistent and cross-reactive antibody responses after vaccination with squalene-based adjuvants^56–59^. Thus, both the antigen and adjuvant in S-268019-b could contribute to sustained and broad anti-RBD antibody responses after the heterologous booster. A recent study has demonstrated that S-268019-b booster vaccination enhances neutralization breadth against SARS-CoV-2 Omicron subvariants including BA.2.12.1, BA.4, and BA.5 in cynomolgus macaques^30^, which strongly supports our results. Similar to the S-268019-b booster, another group has also recently reported that, although there are no clear differences in memory B cell responses, a heterogeneous booster with Ad26.COV2.S adenovirus vector vaccine induces more durable BA.1-neutralizing antibody responses in BNT162b2 vaccinees than the BNT162b2 homologous booster^60^. However, it remains unclear whether the differences in the durability and breadth of immune responses among different vaccine modalities are due to differences in the dose of antigen, differences in the kinetics of antigen expression in vivo, subtle differences in antigen structure, and/or differences in the pattern of antigen presentation/recognition. To clarify these issues, further investigation into the detailed mechanism of the heterologous booster by S-268019-b and other modality vaccines will be necessary.

Identifying biomarkers associated with vaccine immunogenicity is important for developing effective vaccines and designing the heterologous booster. We have previously shown that the early dynamics of NK/monocyte subsets (CD16^+^ NK cells, CD56^high^ NK cells, and non-classical monocytes) correlated with neutralizing antibody titers after the second dose of the BNT162b2 vaccine^18^. In this study, we again identified the early dynamics of the CD16^+^ NK cells as an immune correlate with the RBD-ACE2 binding inhibitory activities after a heterologous S-268019-b booster. Notably, our data also suggest the possible links between CCR3 expression on the CD16^+^ NK cells and sustained and broad anti-RBD antibody responses. The functional roles of the CCR3 in the NK cells have not yet been reported. CCR3 is expressed on many cell types, including eosinophils, basophils, mast cells, and Th2-type lymphocytes, and it regulates Th2-type immune responses^61,62^, suggesting that CCR3^+^ NK cell subtypes may present a shift towards anti-inflammatory Th2-type cells (e.g. IL-10-producing NK cells^63^) rather than pro-inflammatory Th1-type cells. CCR3 plays a role in cell migration induced by multiple CCR3 ligands^61,62^. After the S-268019-b booster, low but detectable levels of Th2-type immune responses are elicited^29^, which may be involved in the unusual CCR3 expression on the CD16^+^ NK cells even without systemic symptom scores. Besides cell migration, CCR3 also promotes the release of cytokines, including leukotriene C4 from eosinophils/ basophils and IL-4 from eosinophils^64,65^. After vaccination, NK cells are recruited into lymph nodes, contributing to efficient T/B cell responses via the upregulated IFN-γ or IL-6^66–68^. The upregulated CCR3 expression on the CD16^+^ NK cells may contribute to the chemotaxis and/or well-balanced cytokine production following a heterologous booster of S-268019-b. It will be intriguing to pursue further the involvement of the CD16^+^ NK cells in the sustained and broad anti-RBD antibody responses without systemic symptoms via the unique CCR3 ligands/CCR3 axis.

## Methods

### Ethics statement

All studies were approved by the Institutional Review Board of the National Institute of Infectious Diseases and Tokyo Shinagawa Hospital (Permit numbers: 1322 and 21-A-11). This study was performed according to the principles of the Declaration of Helsinki.

### Study design

In this investigation, the relationships among AEs, antibody titers to SARS-CoV2 variants, and immune cell dynamics induced by two vaccine modalities were assessed. We recruited 184 Japanese participants who had received two doses of the BNT162b2 mRNA vaccine. Of these participants, 90 received BNT162b2, and 94 received S-268019-b as their third vaccination. The questionnaire on AEs that occurred 0–7 days after the vaccination was completed by all participants. A longitudinal analysis of the plasma RBD-binding antibody and the inhibitory activity on RBD-ACE2 binding against the Wuhan, Beta, and Omicron BA.1 variants was performed. Neutralization activities against the Wuhan, Omicron BA.1, and BA.5 variants were evaluated using plasma on day 68 after the third vaccination. We profiled immune cell and cytokine/chemokine dynamics in the peripheral blood during days 0 and 1 using flow cytometry and a Luminex assay, respectively.

### Human samples

Volunteers in this study (aged ≥20 years) participate in a phase 2/3, randomized, observer-blinded, noninferiority study of S-268019-b recombinant SARS-CoV-2 spike protein vaccine (jRCT2031210470), which is supported by Shionogi & Co., Ltd., and Ministry of Health, Labor and Welfare (MHLW), as previously reported^29^. All participants had received a second dose of the BNT162b2 mRNA vaccine (Pfizer/BioNTech) ≥6 months prior and an intramuscular injection of 0.3 mL of BNT162b2 (30 μg in saline, *n* = 90) or 0.5 mL of S-268019-b (10 μg antigen prepared with 50% v/v oil-in-water adjuvant emulsion) on day 0 as the third vaccination. Longitudinal blood samples were collected on days 0, 1, 14, 28, and 68 after the third vaccination at Tokyo Shinagawa Hospital in Vacutainer CPT tubes (BD Biosciences, #362761). All participants were examined for anti-nucleocapsid antibody titers at pre-vaccination (day 0) and post-vaccination (day 68) and excluded the samples from further analysis if they showed >0.1 antibody titer, which is lower than the 1.0 cut-off recommended by the manufacturer (Roche kit Elecsys® Anti-SARS-CoV-2 RUO, #518316181) and even lower than the cut-off (0.128) described in a previous report^69^. As for the informed consent, all participants provided two separate, written informed consents for the clinical trial (jRCT2031210470) and this accompanying study prior to the enrollment. All participants were notified that the objectives of this study differed from those of the clinical trial, and provided additional blood specifically for this study with their consent.

### Sample processing and cell isolation

Blood samples were collected in Vacutainer CPT tubes (BD Biosciences), and peripheral blood mononuclear cells (PBMCs) and plasma samples were isolated via centrifugation at 1800 × *g* for 20 min. Plasma containing PBMCs was collected into different tubes, followed by centrifugation at 300 × *g* for 15 min. After the plasma was transferred into another tube, the PBMC pellets were washed with phosphate-buffered saline (PBS) (Fujifilm Wako Pure Chemicals, #166-23555) three times before cryopreservation in CELLBANKER 1 plus (ZENOGEN PHARMA, #CD021). The plasma samples were further centrifuged at 800 × *g* for 15 min, transferred into another tube to remove the PBMCs completely, and stored at −80 °C until further analysis. The plasma samples were used immediately after thawing for cytokine/chemokine quantification and heat-inactivated at 56 °C for 30 min before use for RBD-, spike-, and NTD-binding IgG and relative RBD-ACE2 inhibition quantification. According to the manufacturer’s instructions, the nucleocapsid antibody was analyzed using Cobas e411 plus (Roche) with Elecsys Anti-SARS-CoV-2 (Roche).

### Questionnaire on AEs

All participants completed a questionnaire regarding vaccine-related AEs that occurred 0–7 days after receiving their booster vaccination. The questionnaire asked about the presence of eight systemic symptoms (fever, fatigue, headache, chills, vomiting, diarrhea, muscle pain, and joint pain), and symptom severity was rated on a 5-point scale (grade 0-4, see Table S1). The criteria used in the BNT162b2 mRNA vaccine clinical study were applied^19^. The systemic symptom severity scores were calculated as the sum of the eight systemic symptom severity grades.

### Electrochemiluminescence immunoassay

RBD-binding IgG titers and RBD-ACE2 inhibition were measured using the V-PLEX SARS-CoV-2 Panel 22 (IgG) kit (Meso Scale Discovery, #K15559U). For the RBD-binding IgG titer, the V-PLEX Panel 22 plates were supplied with MSD Blocker A reagent, and they were incubated at room temperature for 1 h with rotation for blocking. The plates were then washed with washing buffer [PBS supplemented with 0.05% Tween 20 (Fujifilm Wako Pure Chemicals, #167-11515) three times and incubated with samples diluted in Diluent 100 (Meso Scale Discovery, #R50AA) at room temperature for 2 h with rotation]. The plates were washed three times and then incubated with SULFO-TAG-conjugated anti-human IgG (1:200, contained in the kit #K15559U, Meso Scale Discovery) at room temperature for 1 h with rotation. The plates were then washed again with the washing buffer and filled with MSD Gold read buffer B (Meso Scale Discovery, #R60AM), and electrochemiluminescence was immediately measured with MESO QuickPlex SQ120 (Meso Scale Discovery, #AI0AA). RBD-binding IgG titers were calculated using a reference standard (Meso Scale Discovery) followed by conversion to WHO/NIBSC international units (BAU/mL).

For the RBD-ACE2 inhibition assay, the Panel 22 plates were blocked with the blocking buffer for 1 h, washed with the washing buffer, and incubated for 1 h with plasma diluted by 1/10 times. After incubating, SULFO-TAG Human ACE2 Protein (Meso Scale Discovery, #D21ADG) was added without washing, followed by incubation at room temperature for 1 h. The plates were washed with the washing buffer, followed by the addition of MSD Gold read buffer B (Meso Scale Discovery), and electrochemiluminescence was immediately measured using MESO QuickPlex SQ120 (Meso Scale Discovery). ACE2-binding signals were normalized to those obtained in blank wells (only the diluent was added instead of diluted plasma) for each variant RBD.

The decay rate of antibody titers was calculated as follows: The reduction of antibody titers was first calculated by subtracting the peak antibody titer (T_Peak_) from the antibody titer on day 68 (T_68_), i.e., (T_68_-T_Peak_). The peak antibody indicates a higher titer on day 14 or 28. The reduction ratio was calculated by dividing (T_68_-T_Peak_) by T_Peak_. Finally, the reduction rate/day was calculated by the number of days between day 68 and the peak time point. For the decay rate analysis, the reduction rate above 0 was defined as 0. Individuals with a decay rate <0 were defined as decayers and individuals with a decay rate of 0 as sustainers.

### Enzyme-linked immunosorbent assay

Maxisorp 384 well plates (Nunc, #P6366) were coated with 1 µg/mL of Wuhan NTD (amino acids 1 to 162 of spike) or trimeric Wuhan spike proteins prepared as described previously^70^ at 4 °C overnight. After washing with wash buffer (PBS supplemented with 0.05% Tween-20), the plates were blocked with 1% BSA (Roche, #10735086001)/PBS at room temperature for 1 h. After washing with the wash buffer, the plates were incubated with serially diluted plasma (6-fold serial dilutions starting from 1:500) in Diluent 100 (Meso Scale Discovery) for 2 h at room temperature. The plates were washed with the wash buffer and incubated with HRP-conjugated goat anti-human IgG (1:5000, polyclonal, Southern Biotech, #2040-05) for 1 h at room temperature. After washing with the washing buffer, HRP activity was visualized with OPD substrate (Sigma, #523121), followed by the addition of HCl to stop the reaction. Absorbance at 490 nm was measured using Epoch2 (Biotek). Binding curves were analyzed with GraphPad Prism software with 4-parameter nonlinear regression to calculate the EC_50_ values.

### Pseudotyped virus neutralization assay

Wuhan, Omicron BA.1, and BA.5 SARS-CoV-2 pseudotyped viruses were generated as described previously^71^. The pCAGGS expression vector was generated with cDNA encoding the spike proteins of the SARS-CoV-2 viruses, including WK-521 (Wuhan), TY38-873 (hCoV-19/Japan/TY38-873P0/2021, Omicron BA.1), and TY41-702 (hCoV-19/Japan/TY41-702P0/2022, Omicron BA.5), and the plasmid (pCAG-SARS-CoV-2) containing a 19 aa truncation at the C-terminus of the spike protein was constructed. The expression vector was transfected into 293 T cells (American Type Culture Collection, #CRL-3216) on collagen-coated tissue culture plates. After incubating for 24 h, the cells were infected with G-complemented VSVΔG/Luc^72^ at a multiplicity of infection of 0.5. After 24 h of incubation, VSV pseudotyped viruses were collected from the centrifuged culture supernatants and stored at −80 °C. For pseudotyped virus neutralization (PVNT) assay, VeroE6/TMPRSS2 cells (JCRB Cell Bank, #JCRB1819) were maintained in DMEM (Fujifilm Wako Pure Chemical, #044-29775) containing 10% heat-inactivated fetal bovine serum (Biowest, #S1780-500), 1 mg/mL geneticin (Thermo Fisher Scientific, #10131-027), and 100 U/mL penicillin/streptomycin (Thermo Fisher Scientific, #15140-122). SARS-CoV-2 pseudotyped viruses were incubated with heat-inactivated plasma samples (five 6-fold serial dilutions starting at 1:10 dilution) for 1 h at 37 °C. After the incubation, the mixture was inoculated into VeroE6/TMPRSS2 cells seeded in 96-well solid white flat-bottom plates (Corning, #3917), and then incubated for 24 h at 37 °C with 5% CO_2_. Luciferase activity in cultured cells was measured using the Bright-Glo Luciferase Assay System (Promega, #E2610) with a GroMax Navigator Microplate Luminometer (Promega, #GM2000). Half-maximal inhibitory concentration (IC_50_) was calculated using Prism 9 (GraphPad) as PVNT titers. PVNT titers under the detection limit (IC_50_ = 20) were set to 20 and the detection limit was indicated as a dotted line.

### Flow cytometry

Antigen probes were prepared to analyze memory B cells as described previously^73^. Biotinylated Wuhan spike, Wuhan-RBD, Beta, and Omicron (BA.1)-RBD were mixed with streptavidin-phycoerythrin (PE; Invitrogen, #12431787), streptavidin-PE-CF594 (BD Biosciences, #562284), streptavidin-PE/Cyanin5 (BD Biosciences, #554062), and allophycocyanin (APC; Invitrogen, #17-4317-82), respectively, at a 4:1.5 ratio and incubated at 4 °C overnight, followed by addition of 10 µM biotin (Tokyo Chemical Industry, #58-85-5). For staining, PBMCs were thawed at 37 °C and immediately washed with B cell media [RPMI 1640 (Fujifilm Wako Pure Chemical Corporation, #189-02025) supplemented with 10% FCS HyClone (Thermo Fisher Scientific, #30071.03), 2-mercaptoethanol (Gibco, #21985023), 100 U/mL penicillin (Gibco, #15140122), 100 μg/mL streptomycin (Gibco, #15140122), 10 mM HEPES (Gibco, #15630080), 1 mM Sodium Pyruvate (Gibco, #11360070), and 1% MEM non-essential amino acids solution (Gibco, #11140050)] followed by incubation with the spike/RBD probes [Wuhan spike-PE, 1:100 (final dilution factors are indicated hereafter); Wuhan RBD-PE-CF594 or BUV661, 1:200; Beta-RBD-PE/Cy5, 1:100, Omicron (BA.1)-RBD-APC, 1:50] and streptavidin-PE/Cyanine7 (1:100; BioLegend, #405206) as a decoy in the B cell media supplemented with 10 µM biotin for 30 min at room temperature. The cell suspensions were further supplemented with aliquots of B cell media containing antibodies/reagents and the Brilliant Stain Buffer Plus for 30 min at room temperature: anti-IgA-FITC [polyclonal rabbit F(ab′)2, 1:200; Dako, #F0316], anti-IgG-BV421(G18-145, 1:100; BD Biosciences, #562581), anti-human CD2-Brilliant Violet 510 (RPA-2.10, 1:100; BioLegend, #300218), anti-human CD4-Brilliant Violet 510 (RPA-T4, 1:100; BioLegend, #300546), anti-human CD10-Brilliant Violet 510 (HI10a, 1:100; BioLegend, #312220), anti-human CD14-Brilliant Violet 510 (M5E2, 1:100; BioLegend, #301842), LIVE/DEAD Fixable Yellow Dead Cell Stain kit (1:200; Thermo Fisher Scientific, #L34959), anti-CD19-BUV395 (HIB19, 1:100; BD Biosciences, #740287), anti-CD20-BUV496 (2H7, 1:100; BD Biosciences, #749954), anti-IgM-BUV563 (UCH-B1, 1:300; BD Biosciences, #748929), and anti-human IgD-BUV737 (IA6-2, 1:100; BD Biosciences, #612798). The cells were washed with B cell medium, resuspended in the medium, and analyzed using FACSymphony S6 (BD Biosciences) and FACSDiva v.9.1.2 software (BD Biosciences) or Symphony A3 cytometer (BD Biosciences) and FACSDiva v.9.1 software (BD Biosciences). Data were analyzed using the FlowJo software (BD Biosciences).

For analysis of 13 immune cells, PBMCs were thawed at 37 °C and washed twice with RPMI 1640 (Fujifilm Wako Pure Chemical Corporation) containing 10% heat-inactivated fetal bovine serum (Nichirei Biosciences, #171012), 2 mM glutamine (Fujifilm Wako Pure Chemical Corporation), 100 U/mL penicillin (Fujifilm Wako Pure Chemical Corporation, #168-23191), and 100 μg/mL streptomycin (Fujifilm Wako Pure Chemical Corporation, #168-23191) before use. After blocking non-specific antibody binding using Human TruStain FcX (BioLegend, #422402) for 5 min at room temperature, the cells were stained for 2 h at 4 °C with the following antibodies using the Brilliant Stain Buffer Plus (BD Biosciences, #566385): anti-CD1c-BUV737 (F10/21A3, 1:300; BD Biosciences, #748723), anti-CD3-BUV661 (HIT3a, 1:300; BD Biosciences, #741596), anti-CD5-BV480 (UCHT2, 1:300; BD Biosciences, #566122), anti-CD11c-APC-R700 (BU15, 1:300; BD Biosciences, #566875), anti-CD14-BV421 (M5E2, 1:300; BD Biosciences, #565283), anti-CD16-BUV395 (3G8, 1:300; BD Biosciences, #563785), anti-CD19-BV570 (HIB19, 1:150; BioLegend, #302236), anti-CD45-BUV805 (HI30, 1:150; BD Biosciences, #612891), anti-CD56-BB515 (B159, 1:300; BD Biosciences, #561358), anti-CD69-BV711 (FN50, 1:300; BD Biosciences, #563836), anti-CD86-PE (IT.2, 1:50; BD Biosciences, #305406), anti-CD88-PE-Cyanine7 (S5/1, 1:300; BioLegend, #344308), anti-CD123-BUV496 (6H6, 1:300; BD Biosciences, #751836), anti-CD141-BV605 (1A4, 1:300; BD Biosciences, #740421), anti-CD163-PE-CF594 (GHI/61, 1:300; BD Biosciences, #562670), anti-Axl-BUV615 (108724, 1:300; BD Biosciences, #751050), anti-HLA-DR-APC-H7 (G46-6, 1:300; BD Biosciences, #561358), anti-Siglec-6-BV650 (767329, 1:300; BD Biosciences, #747911), anti-CCR1-PE (5F10B29, 1:300; BioLegend, #362904), anti-CCR2-Brilliant Violet 785 (K036C2, 1:300; BioLegend, #150621), anti-CCR3-Brilliant Violet 711 (5E8, 1:300; BioLegend, #310731), anti-CCR5-BV750 (3A9, 1:300; BD Biosciences, #747475), anti-CCR10-APC (1B5, 1:300; BD Biosciences, #564771), anti-CXCR1- PE/Cyanine5 (8F1/CXCR1, 1:300; BioLegend, #320610), anti-CXCR2-BUV563 (6C6, 1:150; BD Biosciences, #749072), anti-CXCR3-PE/Cyanine5 (G025H7, 1:300; BioLegend, #353756), anti-CXCR4-Brilliant Violet 785 (12G5, 1:150; BioLegend, #306530), and anti-IL-18R1-APC (H44, 1:300; BioLegend, #313814). Subsequently, the cells were washed twice and resuspended in PBS containing 0.5% BSA (Sigma-Aldrich, #P8287), 5 mM EDTA (Thermo Fisher Scientific, #15575), and 0.25 μg/mL 7-AAD (Sigma, #A9400) to detect dead cells. Thirteen immune cells were analyzed using a FACSymphony A3 cytometer (BD Biosciences) and FACSDiva v.9.1 software (BD Biosciences).

### Luminex assay

The Bio-Plex Pro Human Cytokine Screening Panel, 48-Plex (#12007283), was purchased from Bio-Rad and used according to the manufacturer’s instructions. Beads were added to a 96-well plate and washed in a Bio-Plex Pro Washer Station. Samples were added to the plate containing the mixed antibody-linked beads and incubated for 1 h at room temperature. Room temperature incubation steps were performed on an orbital shaker at 500–600 rpm. Following the incubation, plates were washed in a Bio-Plex Pro Washer Station and incubated with a biotinylated detection antibody for 30 min at room temperature under shaking conditions. The plates were washed as described above, and streptavidin-PE was added. After incubation for 10 min at room temperature, a wash was performed as described previously, and assay buffer was added to the wells. Each sample was assayed in singlets. Plates were read on a Bio-Plex 3D instrument with a lower bound of 50 beads per sample per cytokine/chemokine. Data were analyzed with Luminex xPONENT for FLEXMAP3D software v.4.3 (Luminex).

### Statistical analysis

Numerical data were statistically analyzed and visualized using GraphPad Prism 9 software (GraphPad). Matched multiple data were analyzed using the Friedman test. Unpaired data were analyzed using the two-tailed Mann–Whitney test. Two categorical variables were analyzed using the Fisher’s exact test. For multiple comparisons after the analyses, the Dunn’s multiple comparisons test was performed. Differences were considered significant at *P* < 0.05: *P < 0.05, **P < 0.01, ***P < 0.001, and ****P < 0.0001. “*n*” indicates the number of biological replicates.

### Reporting summary

Further information on research design is available in the Nature Portfolio Reporting Summary linked to this article.

## Supplementary information

Supplementary Information

Reporting Summary

## Data Availability

All data are included in this article and Supplementary Information. Source Data underlying figures are provided as a Source Data file. Any additional information required to reanalyze the data reported in this paper is available from the corresponding authors upon request. Source data are provided with this paper.

## Source data

Source Data

## Code availability

This paper does not report the original code.
