# Supplementary material for: Heterologous SARS-CoV-2 spike protein booster elicits durable and broad antibody responses against the receptor-binding domain
Source: Nat Commun. 2023 Mar 15;14:1451. doi: 10.1038/s41467-023-37128-1 (PMC10016167; doi:10.1038/s41467-023-37128-1)
Supplement: Supplementary file 1 — Supplementary Information [file 41467_2023_37128_MOESM1_ESM.pdf]

Supplementary Information

Heterologous SARS-CoV-2 spike protein booster elicits durable and broad antibody responses against the receptor-binding domain

Tomohiro Takano, Takashi Sato, Ryutaro Kotaki, Saya Moriyama, Shuetsu Fukushi, Masahiro Shinoda, Kiyomi Kabasawa, Nagashige Shimada, Mio Kousaka, Yu Adachi, Taishi Onodera, Kazutaka Terahara, Masanori Isogawa, Takayuki Matsumura, Masaharu Shinkai, and Yoshimasa Takahashi

Contents

Supplementary Figures 1-6

Supplementary Table 1

Supplementary Figure 1

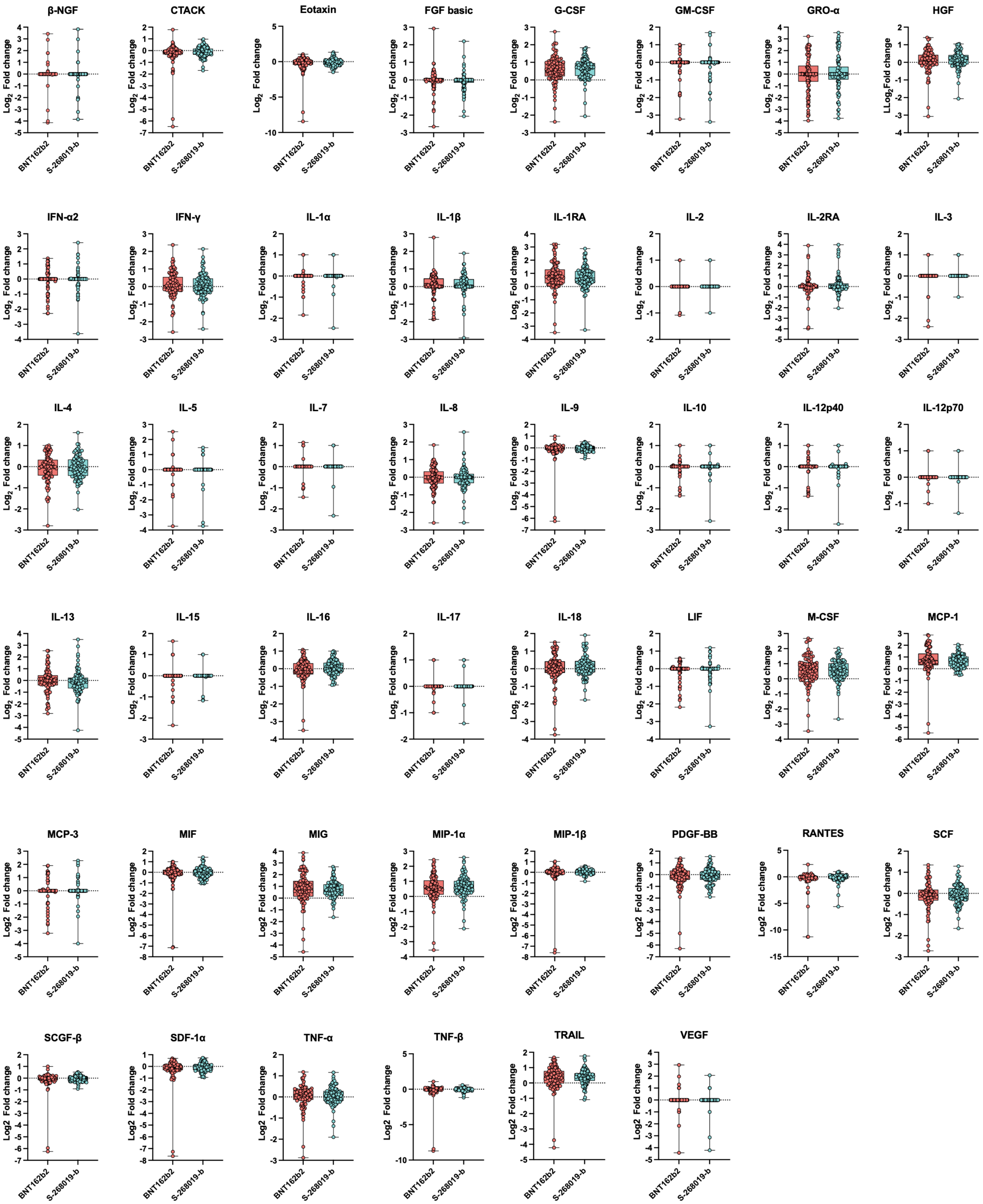

Supplementary Figure 1 | Plasma cytokine/chemokine dynamics following the third vaccination.

Fold change (Day 1 concentration/Day 0 concentration) of the plasma cytokines/chemokines after the third vaccination. For box and whisker plots, the whisker indicates minimum and maximum, and the box indicates the 25th and 75th percentiles (edges of the box), and median (center line). Each dot represents data from individuals. Statistical analyses were performed using the two-tailed Mann-Whitney test. Data were pooled from 3 independent experiments [BNT162b2 (red), n = 90; S-268019-b (blue), n = 94]. Source data are provided as a Source Data file.

Supplementary Figure 2

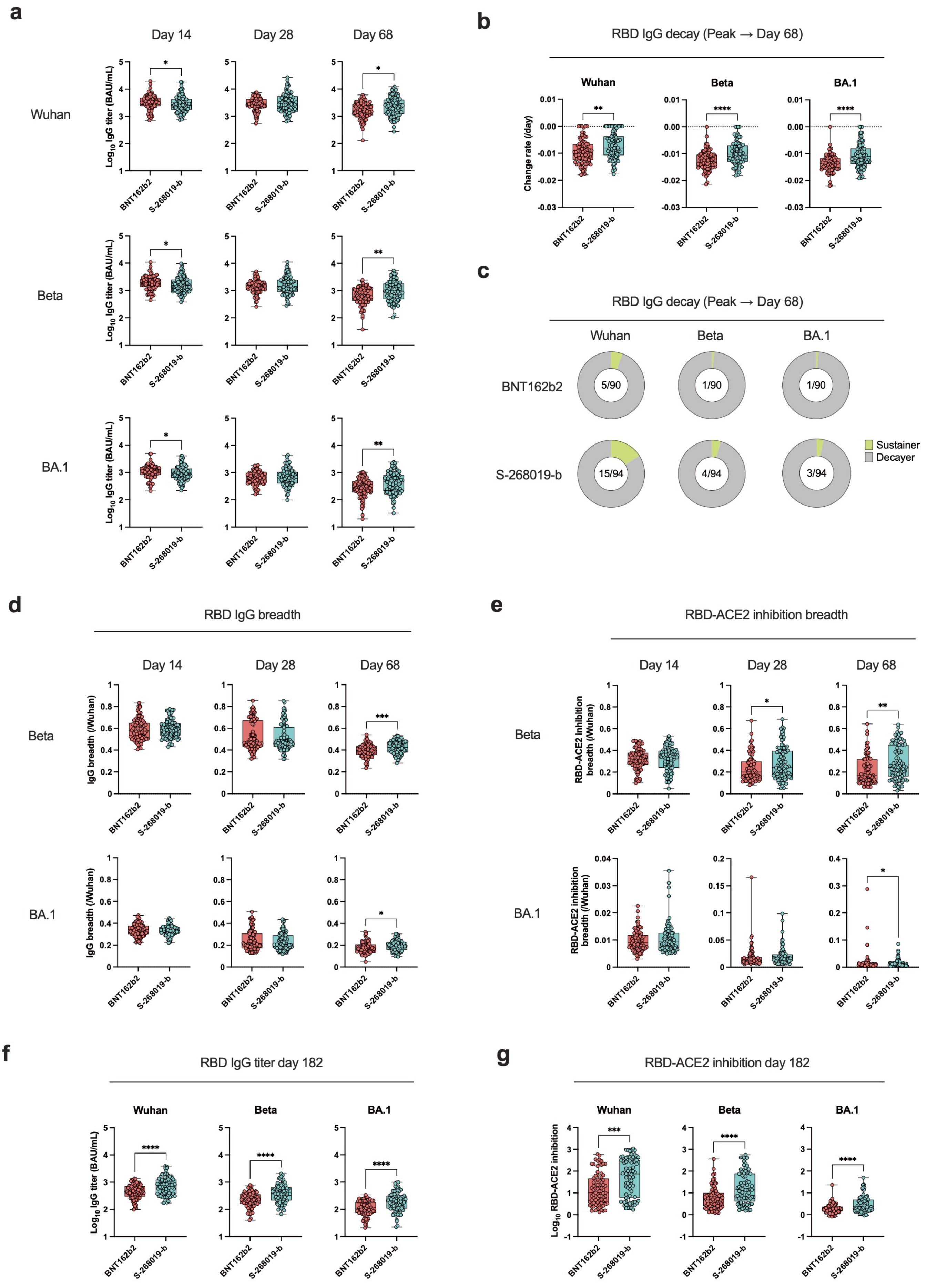

Supplementary Figure 2 | Durability of plasma antibody responses following the third vaccination .

a Receptor-binding domain (RBD)-binding IgG titers for the variants on days 14, 28, and 68. **b** Decay rate of the RBD-binding IgG titers from the peak to day 68 after the third vaccination to the variant RBDs. **c** Ratios of the sustainers and decayers for RBD-binding IgG titer. The breadth of the RBD-binding IgG titers (**d**) and relative RBD-angiotensin-converting enzyme 2 (ACE2) inhibition (**e**) to the variant RBDs on days 14, 28, and 68. **f** RBD-binding IgG titers for the variants on day 182. **g** Relative RBD-ACE2 inhibition to the variants on day 182. For box and whisker plots, the whisker indicates minimum and maximum, and the box indicates the 25th and 75th percentiles (edges of the box), and median (center line) (**a**, **b**, and **d–g**). Each dot represents data from individuals (**a**, **b**, and **d–g**). Statistical analyses were performed using a two-tailed Mann-Whitney test (**a**, **b**, and **d–g**; $*P < 0.05$, $**P < 0.01$, $***P < 0.001$, and $****P < 0.0001$). Data were pooled from ≥ 2 independent experiments [(**a**, **b**, **d**, and **e**) BNT162b2 (red), n = 90; S-268019-b (blue), n = 94; (**f** and **g**) BNT162b2 (red), n = 80; S-268019-b (blue), n = 81]. Source data are provided as a Source Data file.

Supplementary Figure 3

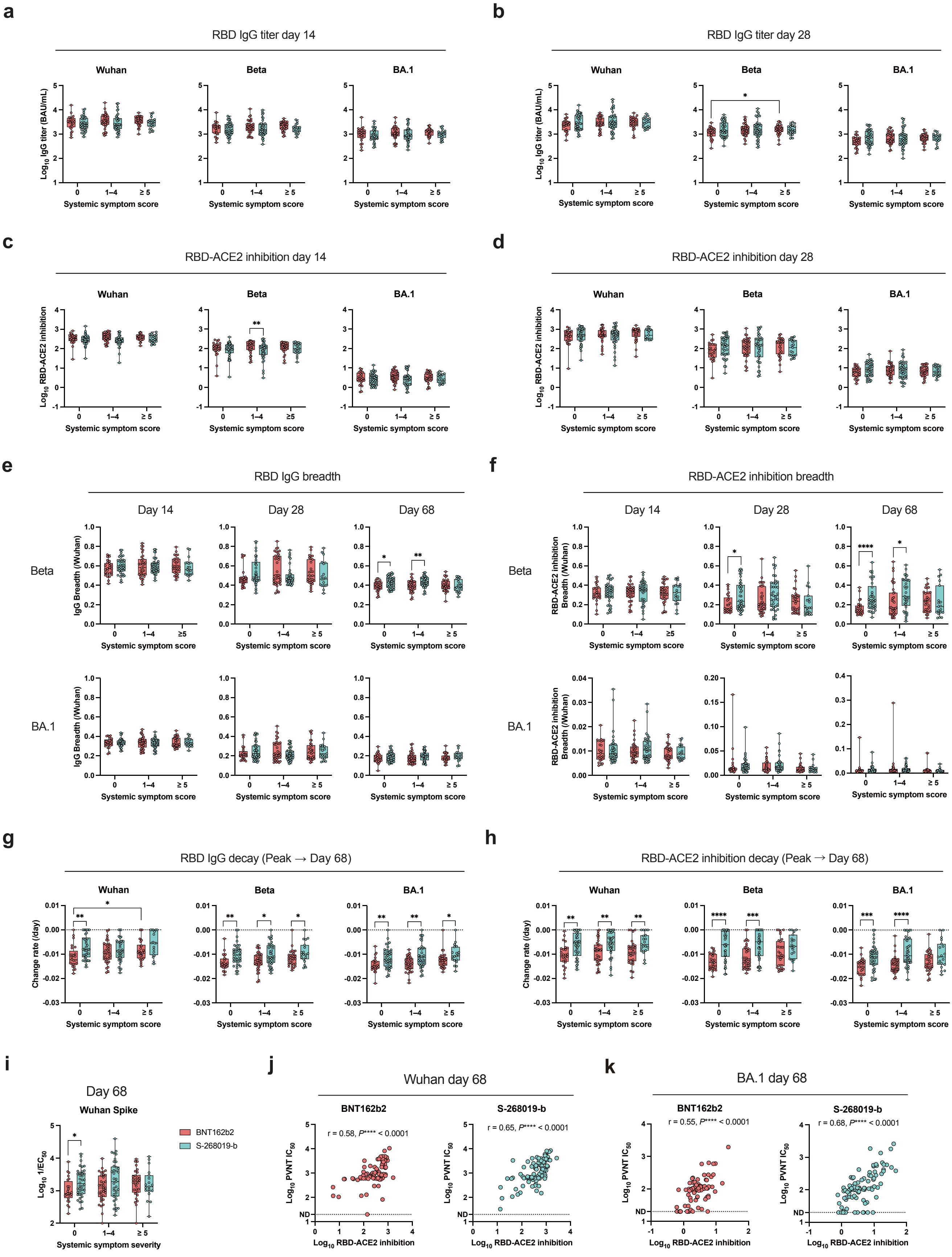

Supplementary Figure 3 | Comparative analyses of the variant reactivity and durability of antibody stratified by the systemic symptom scores.

Receptor-binding domain (RBD)-binding IgG titers for the variant RBDs on days 14 (**a**) and 28 (**b**). Relative RBD-angiotensin-converting enzyme 2 (ACE2) inhibition for the variant RBDs on days 14 (**c**) and 28 (**d**)**.** Breadth of the RBD-biniding IgG titer (**e**) and relative RBD-ACE2 inhibition (**f**) for the variants on days 14, 28, and 68. Decay rate of the RBD-binding IgG titer (**g**) and relative RBD-ACE2 inhibition (**h**) to the variants from the peak to day 68 after the third vaccination. **i** Wuhan spike-binding IgG EC_{50} on day 68. Correlations between RBD-ACE2 inhibition and PVNT IC_{50} for Wuhan (**j**) and Omicron BA.1 (**k**) on day 68. For box and whisker plots, the whisker indicates minimum and maximum, and the box indicates the 25th and 75th percentiles (edges of the box), and median (center line). Each dot represents data from individuals. Statistical analyses were performed using the Kruskal-Wallis test followed by Dunn's post hoc test (comparison among the groups stratified by systemic symptom severity scores within the same vaccine group), two-tailed Mann-Whitney test [comparison between BNT162b2 (red) and S-268019-b (blue) groups within the same systemic symptom severity)(**a–i**), and Spearman's rank order coefficient (**j** and **k**) (*$P < 0.05$, **$P < 0.01$, ***$P < 0.001$, and ****$P < 0.0001$). Data were pooled from ≥ 2 independent experiments [(**a–i)** BNT162b2: n = 25 (score 0), n = 35 (score 1–4), and n = 30 (score ≥ 5); S-268019-b: n = 38 (score 0), n = 37 (score 1–4), and n = 19 (score ≥ 5), (**j** and **k**) BNT162b2: n = 90; S-268019-b: n = 94]. Source data are provided as a Source Data file.

Supplementary Figure 4

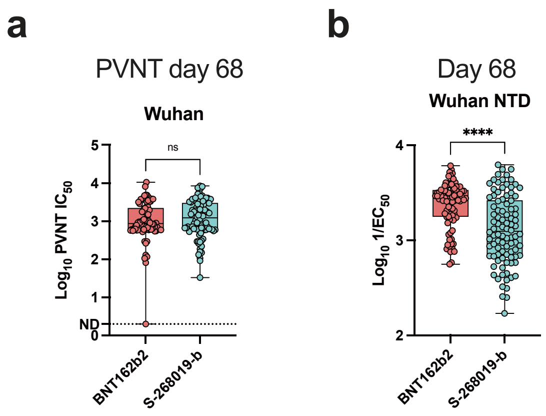

Supplementary Figure 4 | Plasma neutralization activities and anti-NTD antibody responses following the third vaccination.

Neutralization half-maximal inhibition concentration (IC_{50}) (**a**) and Wuhan N-terminal domain (NTD)-binding IgG half-maximal effective concentration (EC_{50}) (**b**) on day 68. For box and whisker plots, the whisker indicates minimum and maximum, and the box indicates the 25th and 75th percentiles (edges of the box), and median (center line). Each dot represents data from individuals. Statistical analyses were performed using a two-tailed Mann-Whitney test (****$P < 0.0001$). Data were pooled from ≥ 2 independent experiments [BNT162b2 (red), n = 90; S-268019-b (blue), n = 94). Source data are provided as a Source Data file.

Supplementary Figure 5

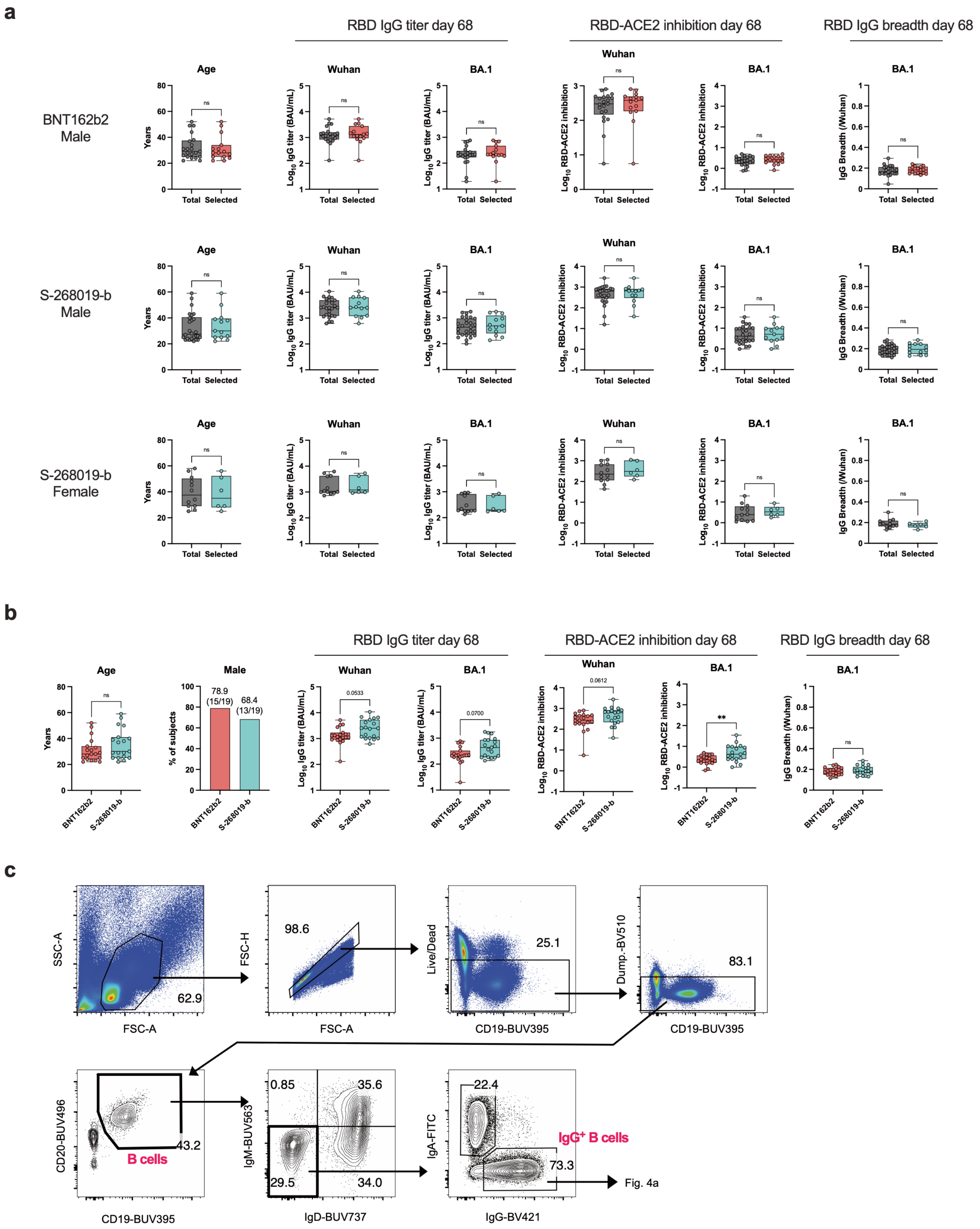

Supplementary Figure 5 | Demographic and antibody reactivities in the participants selected for the B_{mem} analysis.

a Age, receptor-binding domain (RBD)-binding IgG titers, relative RBD-angiotensin-converting enzyme 2 (ACE2) inhibition, and breadth of RBD-binding IgG titers against the variants on day 68 were compared between the total cohort (gray) (for each vaccine and gender group) and the selected samples (BNT162b2: red or S-268019-b: blue) for memory B cell subset analysis. For females in the BNT162 b2 groups without systemic adverse events (AEs) were all analyzed in Fig. 4. **b** Age, frequency of male, RBD-binding IgG titers, relative RBD-ACE2 inhibition, and breadth of the RBD-binding IgG titers against the variants were compared between the BNT162b2 and S-268019-b groups selected for the memory B cell subset analysis. **c** Representative gating strategy for IgG^+ B cells in peripheral blood. Dump: CD2, CD4, CD10, and CD14. For box and whisker plots, the whisker indicates minimum and maximum, and the box indicates the 25th and 75th percentiles (edges of the box), and median (center line). Each dot represents data from individuals. Statistical analyses were performed using the two-tailed Mann-Whitney test (*$P < 0.05$ and **$P < 0.01$). Data were pooled from ≥ 2 independent experiments [**a** BNT162b2 Male: n = 21 (Total), n = 15 (Selected); S-268019-b Male: n = 26 (Total), n = 13 (Selected); S-268019-b Female: n = 12 (Total), n = 6 (Selected); **b** BNT162b2: n = 19; S-268019-b, n = 19]. Source data are provided as a Source Data file.

Supplementary Figure 6

a

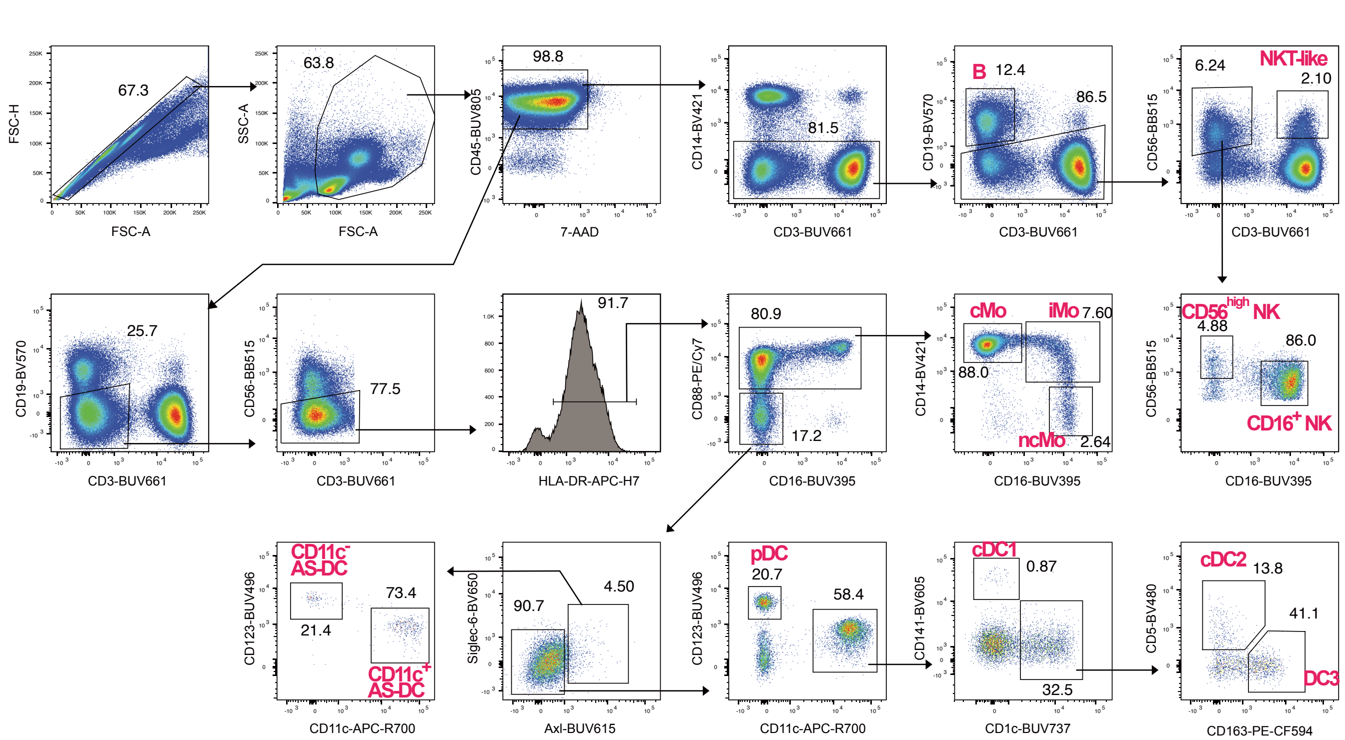

b

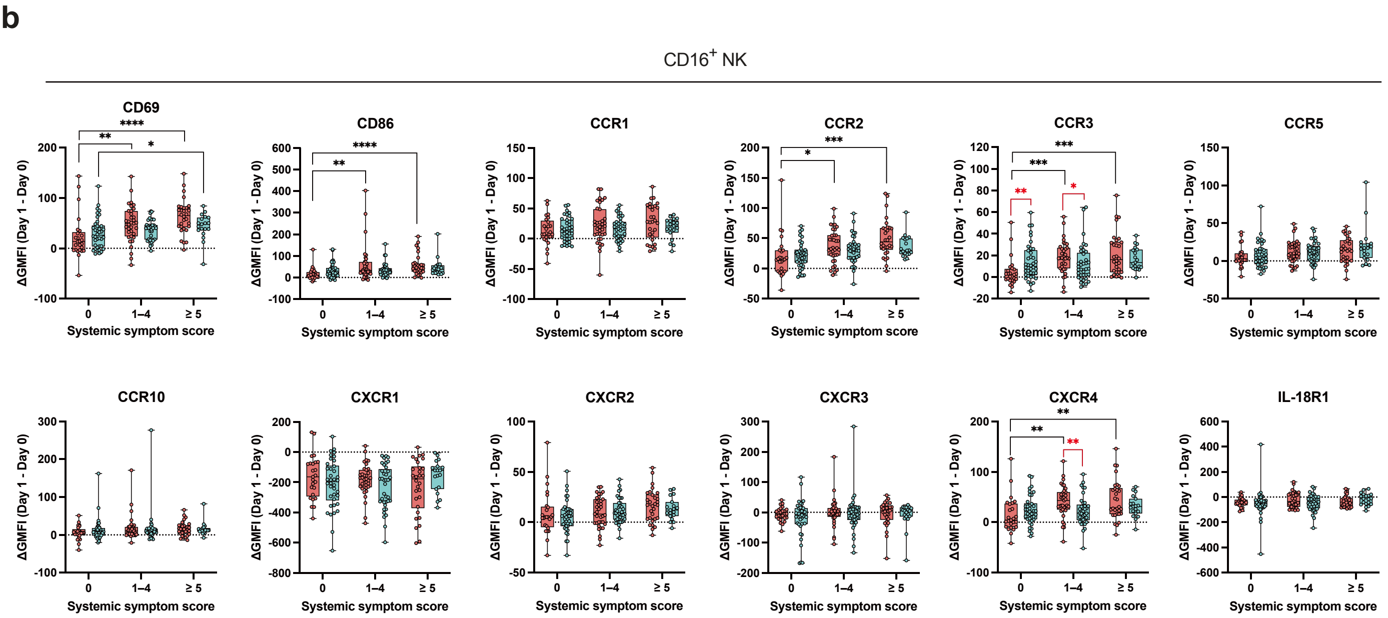

Supplementary Figure 6 | Flow cytometric analysis of the early immune cell dynamics.

a Gating strategy for identifying B cells, CD16^{+} NK cells, CD56^{high} NK cells, NKT-like cells, classical monocytes, intermediate monocytes, non-classical monocytes, conventional DC1 (cDC1s), cDC2s, DC3s, CD11c^{+} AS-DCs, CD11c^{-} AS-DCs, and pDCs. **b** Differences in the CD69, CD86, CCR1, CCR2, CCR3, CCR5, CCR10, CXCR1, CXCR2, CXCR3, CXCR4, and IL-18R1 expression levels on CD16^{+} NK cells between days 1 and 0. For box and whisker plots, the whisker indicates minimum and maximum, and the box indicates the 25th and 75th percentiles (edges of the box), and median (center line). Each dot represents data from individuals (**b**). Statistical analyses were performed using the Kruskal-Wallis test followed by Dunn′s post hoc test (comparison among the groups stratified by systemic symptom severity scores within the same vaccine group) and two-tailed Mann-Whitney test (comparison between BNT162b2 and S-268019-b groups within the same systemic symptom severity) (*$P < 0.05$, **$P < 0.01$, ***$P < 0.001$, and ****$P < 0.0001$). Data were pooled from 6 independent experiments [**b** BNT162b2 (red): n = 25 (score 0), n = 35 (score 1–4), and n = 30 (score ≥ 5); S-268019-b (blue): n = 38 (score 0), n = 37 (score 1–4), and n = 19 (score ≥ 5)]. Source data are provided as Source Data file.

Supplementary Table 1. Criteria of the symptom severity scores.

Systemic Symptom	Grade 1	Grade 2	Grade 3	Grade 4
Fever	38.0 – 38.4 °C	38.5 – 38.9 °C	39.0 – 40 °C	> 40 °C
Fatigue	No interference with activity	Some interference with activity	Prevents daily activity	ER visit or hospitalization
Headache	No interference with activity	Some interference with activity	Prevents daily activity	ER visit or hospitalization
Chills	No interference with activity	Some interference with activity	Prevents daily activity	ER visit or hospitalization
Vomiting	1 – 2 episodes / 24 h	> 2 episodes / 24 h	Requires intravenous hydration	ER visit or hospitalization
Diarrhea	2 – 3 loose stools / 24 h	4 – 5 loose stools / 24 h	6 or more loose stools / 24 h	ER visit or hospitalization
Muscle pain	No interference with activity	Some interference with activity	Prevents daily activity	ER visit or hospitalization
Joint pain	No interference with activity	Some interference with activity	Prevents daily activity	ER visit or hospitalization
