# Supplementary material for: Heterologous SARS-CoV-2 spike protein booster elicits durable and broad antibody responses against the receptor-binding domain
Source: Nat Commun. 2023 Mar 15;14:1451. doi: 10.1038/s41467-023-37128-1 (PMC10016167; doi:10.1038/s41467-023-37128-1)
Supplement: Supplementary file 2 — Reporting Summary [file 41467_2023_37128_MOESM2_ESM.pdf]

Corresponding author(s): Takayuki Matsumura, Masaharu Shinkai, Yoshimasa Takahashi

Last updated by author(s): Feb 27, 2023

Reporting Summary

Nature Portfolio wishes to improve the reproducibility of the work that we publish. This form provides structure for consistency and transparency in reporting. For further information on Nature Portfolio policies, see our Editorial Policies and the Editorial Policy Checklist.

Statistics

For all statistical analyses, confirm that the following items are present in the figure legend, table legend, main text, or Methods section.

n/a	Confirmed	
☐	☒	The exact sample size (*n*) for each experimental group/condition, given as a discrete number and unit of measurement
☐	☒	A statement on whether measurements were taken from distinct samples or whether the same sample was measured repeatedly
☐	☒	The statistical test(s) used AND whether they are one- or two-sided<br>*Only common tests should be described solely by name; describe more complex techniques in the Methods section.*
☐	☒	A description of all covariates tested
☐	☒	A description of any assumptions or corrections, such as tests of normality and adjustment for multiple comparisons
☒	☐	A full description of the statistical parameters including central tendency (e.g. means) or other basic estimates (e.g. regression coefficient) AND variation (e.g. standard deviation) or associated estimates of uncertainty (e.g. confidence intervals)
☐	☒	For null hypothesis testing, the test statistic (e.g. *F*, *t*, *r*) with confidence intervals, effect sizes, degrees of freedom and *P* value noted<br>*Give P values as exact values whenever suitable.*
☒	☐	For Bayesian analysis, information on the choice of priors and Markov chain Monte Carlo settings
☒	☐	For hierarchical and complex designs, identification of the appropriate level for tests and full reporting of outcomes
☐	☒	Estimates of effect sizes (e.g. Cohen's *d*, Pearson's *r*), indicating how they were calculated

Our web collection on statistics for biologists contains articles on many of the points above.

Software and code

Policy information about availability of computer code

Data collection	BD FACSDiva v.9.1 (associated with BD FACSymphony A3), BD FACSDiva v.9.1.2 (associated with BD FACSymphony S6).
Data analysis	FlowJo v.10.8.1, Luminex xPONENT v.4.3, Graphpad Prism 9.4.1.

For manuscripts utilizing custom algorithms or software that are central to the research but not yet described in published literature, software must be made available to editors and reviewers. We strongly encourage code deposition in a community repository (e.g. GitHub). See the Nature Portfolio guidelines for submitting code & software for further information.

Data

Policy information about availability of data

All manuscripts must include a data availability statement. This statement should provide the following information, where applicable:

- Accession codes, unique identifiers, or web links for publicly available datasets
- A description of any restrictions on data availability
- For clinical datasets or third party data, please ensure that the statement adheres to our policy

All data are included in this article and Supplementary Information. Source Data underlying figures are included as a Source Data file. Any additional information required to reanalyze the data reported in this paper is available from the corresponding authors upon request.

Human research participants

Policy information about studies involving human research participants and Sex and Gender in Research.

Reporting on sex and gender	The term sex was used. Sex was determined based on self-report. In the BNT162b2 and S-268019-b groups, biological male population was 70.0% and 72.3% of the total population, respectively. Sex differences were not analyzed because the ratio of male to female is not 1:1.
Population characteristics	All volunteers in this observational study (aged ≥ 20 years) have participated in a phase 2/3, randomized, observer-blinded, noninferiority study of S-268019-b recombinant SARS-CoV-2 spike protein vaccine (jRCT2031210470) in Japan. Healthy Japanese adults who had received two doses of BNT162b2 vaccine ≥ 6 months before were additionally boosted by BNT162b2 (n = 90) or S-268019-b (n =94). In the BNT162b2 and S-268019-b groups, the median age was 31 (interquartile range [IQR]: 25–37 years) and 30 years (IQR: 25–39 years), median body mass index (BMI) was 21.5 (IQR: 19.4–24.7) and 22.7 (IQR: 20.7–25.1), male population was 70.0% and 72.3% of the total population, and the prevalence of comorbidities (hypertension, diabetes mellitus, and dyslipidemia) was 3.33% and 4.26%, respectively.
Recruitment	The participants in this study were voluntarily recruited at the Tokyo Shinagawa Hospital. All volunteers provided written informed consent prior to enrollment for this observational study. Scince this is a randomized and observer-blind study, the participants cannot select the type of vaccine. Thus, there is no bias.
Ethics oversight	This study was approved by the Institutional Review Board of the National Institute of Infectious Diseases and Tokyo Shinagawa Hospital (Permit numbers: 1322 and 21-A-11) and was performed according to the principles of the Declaration of Helsinki. All volunteers provided written informed consent prior to enrollment for this observational study.

Note that full information on the approval of the study protocol must also be provided in the manuscript.

Field-specific reporting

Please select the one below that is the best fit for your research. If you are not sure, read the appropriate sections before making your selection.

☒ Life sciences ☐ Behavioural & social sciences ☐ Ecological, evolutionary & environmental sciences

For a reference copy of the document with all sections, see nature.com/documents/nr-reporting-summary-flat.pdf

Life sciences study design

All studies must disclose on these points even when the disclosure is negative.

Sample size	The required number of participants to demonstrate noninferiority of S-268019-b group in GMT and seroresponse rate of SARS-CoV-2 neutralizing antibody on Day 28 compared to BNT162b2 group with at least 80% power at a one-sided significance level of 0.025 was 100 participants per group, and 200 participants in total. At that time, the true GMTR between S-268019-b and BNT162b2 was assumed to be 1.0 and the standard deviation for the log-transformed SARS-CoV-2 neutralizing antibody titer with base 10 was assumed to be 0.4. The seroresponse rate for S-268019-b and BNT162b2 was assumed to be 0.95. Considering that the immunogenicity subset consisting of participants who had a negative anti-SARS-CoV-2 N-protein antibody test result at screening, and that 1 vial of BNT162b2 contained 6 doses, the target number of participants was set at 204 in clinical trial. However, since this study was limited to participants who consented to the observational study, the BNT162b2 group was set at n=90 and the S-268019-b group at n=94.
Data exclusions	No data were excluded from the analyses.
Replication	All the assays were performed once in multiple biological replicates and all the biological replicates are presented. The reproducibility of the results was addressed by performing at least two independent experiments using samples collected from distinct donors. The numbers of independent experiments are indicated in the figure legend section.
Randomization	Eligible participants were randomized 1:1, stratified by age (<40 and ≥40 years) and sex, to receive an intramuscular injection of either BNT162b2 or S-268019-b on day 0. All samples were used for electrochemiluminescence immunoassay, enzyme-linked immunosorbent assay, pseudotyped virus neutralization assay, thirteen immune cells analysis, and luminex assay. However, the memory B cell analysis was performed for available samples in the vaccinees without any systemic symptom scores who were selected without any biases on age, sex, and antibody reactivities (BNT162b2, n = 19; S-268019-b, n = 19).
Blinding	The specimens were anonymized, each specimen was assigned a specific code, and the colleague who performed the experiment handled the coded specimens. Because they handled the coded specimens, they were unable to recognize the vaccination groups. However, data analyses and the experiments for selected samples were conducted in an unblinded way because the investigators were involved in overall conduct of the analyses.

Reporting for specific materials, systems and methods

We require information from authors about some types of materials, experimental systems and methods used in many studies. Here, indicate whether each material, system or method listed is relevant to your study. If you are not sure if a list item applies to your research, read the appropriate section before selecting a response.

Materials & experimental systems

n/a	Involved in the study
☐	☒ Antibodies
☐	☒ Eukaryotic cell lines
☒	☐ Palaeontology and archaeology
☒	☐ Animals and other organisms
☒	☐ Clinical data
☒	☐ Dual use research of concern

Methods

n/a	Involved in the study
☒	☐ ChIP-seq
☐	☒ Flow cytometry
☒	☐ MRI-based neuroimaging

Antibodies

Antibodies used

Fluorochrome, Antibody, Vendor, Catalog#, Clone, Lot#, Dilution rate
APC, CCR10, BD Biosciences, 564771, 1B5, 1194015, 1:300
APC, IL-18R1, Biolegend, 313814, H44, B353133, 1:300
APC-H7, HLA-DR, BD Biosciences, 561358, G46-6, 0220605, 1:300
APC-R700, CD11c, BD Biosciences, 566875, BU15, 1005586, 1:300
BB515, CD56, BD Biosciences, 561358, B159, 0266219, 1:300
BUV395, CD16, BD Biosciences, 563785, 3G8, 0121736, 1:300
BUV395, CD19, BD Biosciences, 740287, HIB19, 1187920, 1:100
BUV496, CD20, BD Biosciences, 749954, 2H7, 118729, 1:100
BUV496, CD123, BD Biosciences, 751836, 6H6, 1048626, 1:300
BUV563, CXCR2, BD Biosciences, 749072, 6C6, 2069485, 1:200
BUV563, IgM, BD Biosciences, 748929, UCH-B1, 1103772, 1:300
BUV615, Axl, BD Biosciences, 751050, 108724, 1039094, 1:300
BUV661, CD3, BD Biosciences, 741596, HIT3a, 1039067, 1:300
BUV737, CD1c, BD Biosciences, 748723, F10/21A3, 1039090, 1:300
BUV737, IgD, BD Biosciences, 612798, IA6-2, 9290545, 1:200
BUV805, CD45, BD Biosciences, 612891, HI30, 1077722, 1:150
BV421, CD14, BD Biosciences, 565283, M5E2, 0064424, 1:300
BV421, IgG, BD Biosciences, 562581, G19-145, 1033053, 1:100
BV480, CD5, BD Biosciences, 566122, UCHT2, 0310849, 1:300
BV510, CD2, Biolegend, 300218, RPA-2.10, B299213, 1:100
BV510, CD4, Biolegend, 300546, RPA-T4, B340177, 1:100
BV510, CD10, Biolegend, 312220, HI10a, B253991, 1:100
BV510, CD14 Biolegend, 301842, M5E2, B291214, 1:100
BV570, CD19, Biolegend, 302236, HIB19, B350345, 1:200
BV605, CD141, BD Biosciences, 740421, 1A4, 1039090, 1:30
BV650, Siglec-6, BD Biosciences, 747911, 767329, 1039081, 1:300
BV711, CCR3, Biolegend, 310731, 5E8, B358452, 1:300
BV711, CD69, BD Biosciences, 563836, FN50, 1162903, 1:300
BV750, CCR5, BD Biosciences, 747475, 3A9, 2069491, 1:300
BV786, CCR2, Biolegend, 357234, K036C2, B300256, 1:200
BV786, CXCR4, Biolegend, 306530, 12G5, B326264, 1:300
FITC, IgA, Dako, F0316, polyclonal rabbit F(ab')2, 20010670, 1:200
PE, CCR1, Biolegend, 362904, 5F10B29, B288277, 1:300
PE, CD86, Biolegend, 305406, IT2.2, B347117, 1:300
PE-CF594, CD163, BD Biosciences, 562670, GHI/61, 0052859, 1:300
PE-Cy5, CXCR1, Biolegend, 320610, 8F1/CXCR1, B337024
PE-Cy5, CXCR3, Biolegend, 353756, G025H7, B342719, 1:300
PE-Cy7, CD88, Biolegend, 344308, S5/1, B304126, 1:300
SULFO-TAG-conjugated anti-human IgG, Meso Scale Discovery, R32AJ, Polyclonal, D00V0019, 1:200
HRP-conjugated goat anti-human IgG, Southern Biothech, 2040-05, Polyclonal G0420-Y170B, 1:5000

Validation

All antibodies used were evaluated by the manufacturers as provided in their websites.
APC, CCR10, BD Biosciences, 564771, https://www.bdbiosciences.com/ja-jp/products/reagents/flow-cytometry-reagents/research-reagents/single-color-antibodies-ruo/apc-mouse-anti-human-ccr10.564771
APC, IL-18R1, Biolegend, 313814, https://www.biolegend.com/ja-jp/search-results/apc-anti-human-cd218a-il-18ralpha-antibody-17486
APC-H7, HLA-DR, BD Biosciences, 561358, https://www.bdbiosciences.com/ja-jp/products/reagents/flow-cytometry-reagents/research-reagents/single-color-antibodies-ruo/apc-h7-mouse-anti-human-hla-dr.561358
APC-R700, CD11c, BD Biosciences, 566875, https://www.bdbiosciences.com/en-us/products/reagents/flow-cytometry-reagents/research-reagents/single-color-antibodies-ruo/apc-r700-mouse-anti-human-cd11c.566875
BB515, CD56, BD Biosciences, 561358, https://www.bdbiosciences.com/en-us/products/reagents/flow-cytometry-reagents/research-reagents/single-color-antibodies-ruo/bb515-mouse-anti-human-cd56-ncam-1.564488
BUV395, CD16, BD Biosciences, 563785, https://www.bdbiosciences.com/ja-jp/products/reagents/flow-cytometry-reagents/research-reagents/single-color-antibodies-ruo/buv395-mouse-anti-human-cd16.563785
BUV395, CD19, BD Biosciences, 740287, https://www.bdbiosciences.com/ja-jp/products/reagents/flow-cytometry-reagents/

research-reagents/single-color-antibodies-ruo/buv395-mouse-anti-human-cd19.740287
BUV496, CD20, BD Biosciences, 749954, https://www.bdbiosciences.com/ja-jp/products/reagents/flow-cytometry-reagents/research-reagents/single-color-antibodies-ruo/buv496-mouse-anti-human-cd20.749954
BUV496, CD123, BD Biosciences, 751836, https://www.bdbiosciences.com/ja-jp/products/reagents/flow-cytometry-reagents/research-reagents/single-color-antibodies-ruo/buv496-mouse-anti-human-il-3r-cd123.751836
BUV563, CXCR2, BD Biosciences, 749072, https://www.bdbiosciences.com/en-ca/products/reagents/flow-cytometry-reagents/research-reagents/single-color-antibodies-ruo/buv563-mouse-anti-human-cd182.749072
BUV563, IgM, BD Biosciences, 748929, https://www.bdbiosciences.com/ja-jp/products/reagents/flow-cytometry-reagents/research-reagents/single-color-antibodies-ruo/buv563-mouse-anti-human-igm.748929
BUV615, Axl, BD Biosciences, 751050, https://www.bdbiosciences.com/ja-jp/products/reagents/flow-cytometry-reagents/research-reagents/single-color-antibodies-ruo/buv615-mouse-anti-human-axl.751050
BUV661, CD3, BD Biosciences, 741596, https://www.bdbiosciences.com/en-ca/products/reagents/flow-cytometry-reagents/research-reagents/single-color-antibodies-ruo/buv661-mouse-anti-human-cd3.741596
BUV737, CD1c, BD Biosciences, 748723, https://www.bdbiosciences.com/en-us/products/reagents/flow-cytometry-reagents/research-reagents/single-color-antibodies-ruo/buv737-mouse-anti-human-cd1c.748723
BUV737, IgD, BD Biosciences, 612798, https://www.bdbiosciences.com/ja-jp/products/reagents/flow-cytometry-reagents/research-reagents/single-color-antibodies-ruo/buv737-mouse-anti-human-igd.612798
BUV805, CD45, BD Biosciences, 612891, https://www.bdbiosciences.com/ja-jp/products/reagents/flow-cytometry-reagents/research-reagents/single-color-antibodies-ruo/buv805-mouse-anti-human-cd45.612891
BV421, CD14, BD Biosciences, 565283, https://www.bdbiosciences.com/ja-jp/products/reagents/flow-cytometry-reagents/research-reagents/single-color-antibodies-ruo/bv421-mouse-anti-human-cd14.565283
BV421, IgG, BD Biosciences, 562581, https://www.bdbiosciences.com/ja-jp/products/reagents/flow-cytometry-reagents/research-reagents/single-color-antibodies-ruo/bv421-mouse-anti-human-igg.562581
BV480, CD5, BD Biosciences, 566122, https://www.bdbiosciences.com/en-ca/products/reagents/flow-cytometry-reagents/research-reagents/single-color-antibodies-ruo/bv480-mouse-anti-human-cd5.566122
BV510, CD2, Biolegend, 300218, https://www.biolegend.com/ja-jp/products/brilliant-violet-510-anti-human-cd2-antibody-12039?GroupID=BLG9913BV510, CD4, Biolegend, 300546, RPA-T4, B340177, 1:100
BV510, CD10, Biolegend, 312220, https://www.biolegend.com/ja-jp/search-results/brilliant-violet-510-anti-human-cd10-antibody-8306
BV510, CD14 Biolegend, 301842, https://www.biolegend.com/ja-jp/products/brilliant-violet-510-anti-human-cd14-antibody-8001
BV570, CD19, Biolegend, 302236, https://www.biolegend.com/ja-jp/products/brilliant-violet-570-anti-human-cd19-antibody-7367
BV605, CD141, BD Biosciences, 740421, https://www.bdbiosciences.com/ja-jp/products/reagents/flow-cytometry-reagents/research-reagents/single-color-antibodies-ruo/bv605-mouse-anti-human-cd14.564055
BV650, Siglec-6, BD Biosciences, 747911, https://www.bdbiosciences.com/en-ca/products/reagents/flow-cytometry-reagents/research-reagents/single-color-antibodies-ruo/bv650-mouse-anti-human-siglec-6-cd327.747911
BV711, CCR3, Biolegend, 310731, https://www.biolegend.com/ja-jp/products/brilliant-violet-711-anti-human-cd193-ccr3-antibody-20622
BV711, CD69, BD Biosciences, 563836, https://www.bdbiosciences.com/ja-jp/products/reagents/flow-cytometry-reagents/research-reagents/single-color-antibodies-ruo/bv711-mouse-anti-human-cd69.563836
BV750, CCR5, BD Biosciences, 747475, https://www.bdbiosciences.com/en-us/products/reagents/flow-cytometry-reagents/research-reagents/single-color-antibodies-ruo/bv750-mouse-anti-human-cd195.747475
BV786, CCR2, Biolegend, 357234, https://www.biolegend.com/ja-jp/products/brilliant-violet-785-anti-human-cd192-ccr2-antibody-17297
BV786, CXCR4, Biolegend, 306530, https://www.biolegend.com/ja-jp/search-results/brilliant-violet-785-anti-human-cd184-cxcr4-antibody-14329?GroupID=BLG1773
FITC, IgA, Dako, F0316, polyclonal rabbit F(ab')2, https://www.agilent.com/store/productDetail.jsp?catalogId=F031601-2
PE, CCR1, Biolegend, 362904, https://www.biolegend.com/ja-jp/products/pe-anti-human-cd191-ccr1-antibody-9954
PE, CD86, Biolegend, 305406, https://www.biolegend.com/ja-jp/products/pe-anti-human-cd86-antibody-778
PE-CF594, CD163, BD Biosciences, 562670, https://www.bdbiosciences.com/ja-jp/products/reagents/flow-cytometry-reagents/research-reagents/single-color-antibodies-ruo/pe-cf594-mouse-anti-human-cd163.562670
PE-Cy5, CXCR1, Biolegend, 320610, https://www.biolegend.com/ja-jp/products/pe-cyanine5-anti-human-cd181-cxcr1-antibody-3003?GroupID=BLG4174
PE-Cy5, CXCR3, Biolegend, 353756, https://www.biolegend.com/ja-jp/products/pe-cyanine5-anti-human-cd183-cxcr3-antibody-21423
PE-Cy7, CD88, Biolegend, 344308, https://www.biolegend.com/ja-jp/products/pe-cyanine7-anti-human-cd88-c5ar-antibody-7710?GroupID=BLG9979
SULFO-TAG-conjugated anti-human IgG, Meso Scale Discovery, R32AJ, https://www.mesoscale.com/ja-JP/products/anti-human-antibody-goat-sulfo-tag-labeled-r32aj/
HRP-conjugated goat anti-human IgG, Southern Biothech, 2040-05, https://www.southernbiotech.com/goat-anti-human-igg-hrp-2040-05

Eukaryotic cell lines

Policy information about cell lines and Sex and Gender in Research

Cell line source(s)

293T cell line, American Type Culture Collection, CRL-3216
VeroE6/TMPRSS2 cell line, JCRB Cell Bank, JCRB1819

Authentication

293T cell line was from ATCC, and no additional authentication was performed.
VeroE6/TMPRSS2 cell line was from JCRB Cell Bank, and no additional authentication was performed.

Mycoplasma contamination

All cell lines were confirmed negative for mycoplasma contamination.

Commonly misidentified lines
(See ICLAC register)

None

Flow Cytometry

Plots

Confirm that:

☒ The axis labels state the marker and fluorochrome used (e.g. CD4-FITC).

☒ The axis scales are clearly visible. Include numbers along axes only for bottom left plot of group (a 'group' is an analysis of identical markers).

☒ All plots are contour plots with outliers or pseudocolor plots.

☒ A numerical value for number of cells or percentage (with statistics) is provided.

Methodology

Sample preparation	PBMCs were isolated from whole blood samples using BD Vacutainer CPT tubes. Flow cytometric analysis was performed on cryopreserved PBMCs.
Instrument	BD FACSymphony S6, BD FACSymphony A3
Software	Data were acquired using BD FACSDiva (v.9.1, or v.9.1.2) and the data were analyzed using FlowJo v.10.8.1.
Cell population abundance	No cell sorting procedure was used in the study.
Gating strategy	CD14- cells were separated and CD19+CD20+ cells were defined as B cells. IgD-IgM- cells were separated from B cells, and IgA-IgG+ cells were defined as IgG B cells. Among IgG B cells, Spike+ IgG B cells were identified as Spike-probe+ Decoy-probe- cells. In Spike+ IgG B cells, RBD+ IgG B cells were identified as Wuhan-RBD-probe+ cells. Among the RBD+IgG B cells, Beta-binding and Beta-BA.1-binding cells were identified as Beta-RBD-probe+ and Beta-BA.1-RBD-probe+ cells, respectively. Detailed gating strategies are shown in Supplementary Figure 5 and Fig. 4.<br>For 13 immune cell analysis, singlets were gated, then total leukocyte and live leukocytes (CD45+7-AAD-) were gated. From live leukocytes, CD14- cells were separated for, B cell, NK cell analysis. B cells were defined as CD3-CD19+ cells. NK cells were separated from CD3-CD19-CD56+ cells, and CD56high NK cells and CD16+ cells were determined using CD56 and CD16. NKT-like cells were defined as CD3+CD56+ cells. For myeloid cell analysis, CD3-CD19-CD56- cells were separated from live leukocyte. From HLA-DR+ cells, monocytes and DCs were separated using CD16 and CD88. In the monocytes population (CD88+ cells), classical monocytes, non-classical monocytes, and intermediate monocytes were separated using CD14 and CD16. In the DC population (CD16-CD88- cells), Axl+Siglec-6+ cells were separated and CD11c+ AS-DC and CD11c- AS-DC were determined. From Axl-Siglec-6- cells, pDCs was defined as CD11c-CD123+ cells, and in CD11c+CD123- cells, cDC1s were identified as CD1c-CD141+ cells. From CD1c+CD141- cells, cDC2s and DC3s were separated using CD5 and CD163. Detailed gating strategies are shown in Supplementary Figure 6.

☒ Tick this box to confirm that a figure exemplifying the gating strategy is provided in the Supplementary Information.
